# BIG3 Inhibits the Estrogen-Dependent Nuclear Translocation of PHB2 via Multiple Karyopherin-Alpha Proteins in Breast Cancer Cells

**DOI:** 10.1371/journal.pone.0127707

**Published:** 2015-06-08

**Authors:** Nam-Hee Kim, Tetsuro Yoshimaru, Yi-An Chen, Taisuke Matsuo, Masato Komatsu, Yasuo Miyoshi, Eiji Tanaka, Mitsunori Sasa, Kenji Mizuguchi, Toyomasa Katagiri

**Affiliations:** 1 Division of Genome Medicine, Institute for Genome Research, Tokushima University, Tokushima, Japan; 2 Department of Orthodontics and Dentofacial Orthopedics, Institute of Biomedical Sciences, Tokushima University Graduate School, Tokushima, Japan; 3 National Institute of Biomedical Innovation, Osaka, Japan; 4 Department of Surgery, Division of Breast and Endocrine Surgery, Hyogo College of Medicine, Hyogo, Japan; 5 Department of Surgery, Tokushima Breast Care Clinic, Tokushima, Japan; Institute of Molecular and Cell Biology, Biopolis, UNITED STATES

## Abstract

We recently reported that brefeldin A-inhibited guanine nucleotide-exchange protein 3 (BIG3) binds Prohibitin 2 (PHB2) in cytoplasm, thereby causing a loss of function of the PHB2 tumor suppressor in the nuclei of breast cancer cells. However, little is known regarding the mechanism by which BIG3 inhibits the nuclear translocation of PHB2 into breast cancer cells. Here, we report that BIG3 blocks the estrogen (E2)-dependent nuclear import of PHB2 via the karyopherin alpha (KPNA) family in breast cancer cells. We found that overexpressed PHB2 interacted with KPNA1, KPNA5, and KPNA6, thereby leading to the E2-dependent translocation of PHB2 into the nuclei of breast cancer cells. More importantly, knockdown of each endogenous KPNA by siRNA caused a significant inhibition of E2-dependent translocation of PHB2 in BIG3-depleted breast cancer cells, thereby enhancing activation of estrogen receptor alpha (ERα). These data indicated that BIG3 may block the KPNAs (KPNA1, KPNA5, and KPNA6) binding region(s) of PHB2, thereby leading to inhibition of KPNAs-mediated PHB2 nuclear translocation in the presence of E2 in breast cancer cells. Understanding this regulation of PHB2 nuclear import may provide therapeutic strategies for controlling E2/ERα signals in breast cancer cells.

## Introduction

Prohibitin 1 and 2 (PHB and PHB2) proteins are highly conserved in eukaryotic cells and exhibit diverse subcellular localization with different functions [1–3]. These molecules are primarily observed in inner mitochondrial membranes via their N-terminal transmembrane domain but are also present in several other localizations such as the cytosol, endoplasmic reticulum, nucleus, and plasma membrane [1]. Both proteins form hetero-oligomeric ring structures in the inner mitochondrial membrane and function as chaperones that maintain mitochondrial integrity and stabilize expression of mitochondrial respiratory enzymes [1–3]. In the nucleus, both proteins are reported to function as transcriptional regulators. In particular, PHB2 is also reported to selectively repress ERα transcriptional activity through its interaction with ERα in the nucleus, indicating that PHB2 acts as a transcriptional co-repressor of ERα [4–7]. However, its subcellular localization remains debated.

Our previous studies identified that brefeldin A-inhibited guanine nucleotide-exchange protein 3 (BIG3) interacts and co-localizes with PHB2 in the cytoplasm of breast cancer cells [8, 9]. Depletion of BIG3 by siRNA leads to the E2-dependent nuclear translocation of the cytoplasmic PHB2, thereby enabling it to interact directly with ERα [8, 9]. Furthermore, we demonstrated that a dominant-negative peptide, ERAP [9], and a natural compound, Xanthohumol (XN) [10], which specifically disrupt the BIG3-PHB2 interaction, leads to the E2-dependent nuclear translocation of PHB2. This enables PHB2 to directly bind ERα and suppress its transcriptional activity [9, 10]. Thus, understanding the regulation of the nuclear translocation of this PHB2 co-repressor is critical to further elucidate the E2 stimulus-dependent cell proliferation of ERα-positive breast cancers. However, the mechanism underlying the E2-dependent nuclear translocation of PHB2 released from BIG3 via ERAP and XN, or siRNA-BIG3 treatment remains unresolved.

Nuclear import of large molecules is generally mediated by nuclear localization signals (NLS), which contain basic amino acids [11, 12]. Two types of NLS have been identified: one consisting of a monopartite sequence of basic amino acids and the other a bipartite sequence of two clusters of basic amino acids [11, 12]. Proteins containing classic NLS (cNLS) are known to be transported into the nucleus by forming complexes with shuttling carriers, such as Karyopherin-alpha and-beta (KPNA and KPNB) heterodimers or KPNB alone [11, 12]. However, in addition to the cNLS-mediated pathway, KPNB was recently demonstrated to function in the absence of KPNAs through a nonclassic NLS [11, 12]. Accordingly, the mechanism recognizing cargo substrates by KPNAs and KPNB remains unclear.

Previous reports have shown that PHB2 has a putative cNLS [4, 13]. However, whether this sequence is crucial for its nuclear translocation has not been addressed. Here we report the mechanism by which BIG3 blocks the nuclear translocation of PHB2 via interactions with multiple karyopherin alpha (KPNA) proteins, including KPNA1, KPNA5, and KPNA6, in ERα-positive breast cancer cells.

## Materials and Methods

### Ethical statement

All experiments in this study were conducted according to protocols reviewed and approved by the Committee for Safe Handling of Living Modified Organisms Permission number 26–93) in the University of Tokushima.

### Cell lines

Human breast cancer cell lines MCF-7, YMB-1, ZR-75-1, SK-BR-3, HCC1937, MDA-MB-453, MDA-MB-157, MDA-MB-231, BT-549, HCC1143 and HCC1395, human embryonic kidney fibroblast HEK293T cells, as well as the African green monkey SV40-transfected kidney fibroblast cell line COS-7, were purchased from the American Type Culture Collection (ATCC, Rockville, MD, USA). The KPL-3C cells were established, characterized and kindly provided by Dr. Jun-ichi Kurebayashi (Kawasaki Medical School) [14]. All of the cell lines were cultured according to the respective depositor’s recommendations. The cell line stocks that were used in this study had been properly stored in liquid nitrogen. We monitored the cell morphology of these cell lines by microscopy and confirmed that they maintained their morphology by comparing images with the original morphologic images as described above. No *Mycoplasma* contamination was detected in any of the cultures using a *Mycoplasma* Detection kit (Roche, Basel, Switzerland).

### Semi-quantitative reverse transcription-PCR analysis

Total RNA from breast cancer cell lines was isolated using the NucleoSpin RNA II system (Takara-Clontech, Sigma, Japan) according to the manufacturer's instructions. Human mammary gland total RNA, which was pooled from one Caucasian female who caused sudden death, was purchased from Takara-Clontech. Each total RNA was reverse transcribed to single-stranded cDNA using oligo (dT) _12–18_ primers with Superscript II reverse transcriptase (Life Technologies, Carlsbad, CA, USA) as described previously [8, 15]. The PCR primer sequences used were 5′-GTGATCTCCTCACGGTCATG-3′ and 5′-CATAGGAGCCTCACACTG-3′ for *KPNA1*; 5′-GCATAATAGAACCGTTGATG-3′ and 5′-AGGAGCCCCATCCTGAAC-3′ for *KPNA2*; 5′-AATATGAAGCACCACCAGATG-3′ and 5′-GACTGAGACATGGCTTGCTTT-3’ for *KPNA3*; 5′-AGTGGCTTACCTTATCCAAC-3’ and 5′-TGTTGGTACATTGGCAGATG-3’ for *KPNA4*; 5’-TCAGGAACAGGCTGTTTGGG-3’ and 5’-TGGGGTCATCTTCTTCTACAC-3’ for *KPNA5*; 5’-CTGGAGAACATCCTGCGGCTT-3’ and 5’-CTCGTGGCTCTGGAGAAACTC-3’ for *KPNA6*; 5’-ATCCAGCTCGTCCACTCTGG-3’ and 5’-CCTCACCAAAGTGCTTCTCG-3’ for *KPNA7*, and 5'-ATTGCCGACAGGATGCAG-3' and 5'-CTCAGGAGGA GCAATGATCTT-3' for *ACTB* as a quantitative control.

### Immunoprecipitation and immunoblot analyses

Immunoprecipitation and immunoblot analyses were performed as previously described [15, 16]. Briefly, cells were lysed with lysis buffer (50 mM Tris-HCl, pH 8.0; 150 mM NaCl, 0.1% NP-40, 0.5% CHAPS) containing 0.1% protease inhibitor cocktail III (Calbiochem, San Diego, CA, USA). The cell lysates were preincubated with normal IgG and rec-Protein G Sepharose 4B (Life Technologies) at 4°C for 3 h. The supernatants were then incubated with antibodies against PHB2 (5 μg), ERα (5 μg) and anti-FLAG M2 agarose (7.5 μg, Mouse monoclonal, A2220, Sigma, St. Louis, MO, USA) at 4°C for 12 h. Next, the antigen–antibody complexes for the PHB2- and ERα-immunoprecipitates were precipitated with rec-Protein G Sepharose 4B at 4°C for 1 h. The immunoprecipitated protein complexes were washed three times with lysis buffer. The immunoprecipitates and the cell lysates were electrophoretically separated, blotted onto a nitrocellulose membrane and blocked with 4% Block Ace solution (Dainippon Pharmaceutical, Osaka, Japan) for 1 h. The blots were then incubated with antibodies against the following proteins: BIG3 (ref. 8) (1:1000); PHB2 (1:1,000, Rabbit polyclonal, ab135642, Abcam, Cambridge, UK); ERα (SP-1; 1:500, Rabbit monoclonal, RM-9101-S0, Thermo Fisher Scientific, Fremont, CA, USA); α/β-tubulin (1:1,000, Rαbbit polyclonal, #2148, Cell Signaling Technology, Danvers, MA, USA); KPNA1 (2A4-1B5; 1:500, Mouse monoclonal, H00003836-M01), KPNA5 (1D2; 1:500, Mouse monoclonal, H00003841-M01), and KPNA6 (1:500, Goat polyclonal, PAB11515) (Abnova, Taipei, Taiwan); lamin B1 (1:100, Mouse monoclonal, SAB1400153); KPNA2 (1:500, Mouse polyclonal, SAB1406067), β-actin (AC-15; 1:5,000, Mouse monoclonal, A1978) and FLAG-tag M2 (1:5,000, Mouse monoclonal, F3165) (Sigma); and anti-HA (3F10; 1:3,000, Rat monoclonal, #11867423001, Roche). After incubation with a horseradish peroxidase-conjugated secondary antibody (1:5,000: anti-mouse IgG-HRP, polyclonal, sc-2005; anti-rat IgG-HRP, polyclonal, sc-2006; 1:1,000: anti-rabbit IgG-HRP, polyclonal, sc-2004; anti-goat IgG-HRP, polyclonal, sc-2020; Santa Cruz Biotechnology) or monoclonal anti-rabbit immunoglobulins-peroxidase antibody (RG-16; 1:5,000, Rabbit monoclonal, A2074, Sigma) for 1 h, the blots were developed using an enhanced chemiluminescence system (GE Healthcare, Buckinghamshire, UK) and were scanned using an Image Reader LAS-3000 mini (Fujifilm, Tokyo, Japan).

### Construction of expression vectors

To construct the expression vectors, each coding sequence was amplified via PCR using KOD-Plus DNA polymerase (Toyobo, Osaka, Japan). The wildtype PHB2 (1–299) (pCAGGSnHC-PHB2), BIG3 (pCAGGSSnH3F-BIG3) and ERα (pCAGGSn3FC-ERα) expression vectors were constructed previously [8]. The PHB2_1-189_, PHB2_190-299_ and PHB2 mutant (R86A, R88A and K89A) expression vectors were constructed using pCAGGSnHC-PHB2 vector as a template for PCR. The primer sets were as follows; 5’-GCTAACCATGTTCATGCCT-3′ (derived from pCAGGSnHC vector) and 5′-CCGCTCGAGAAAGCTCAGCTCTGTGATGG-3′ for PHB2_1-189_, 5′-CGGAATTCATGAGCCGAGAGTACACAGCT-3′ and 5′-CCGCTCGAGTTTCTTACCCTTGATGAGGC-3′ for PHB2_190-299_ (single underlines indicate the recognition sites of the restriction enzymes), 5′-ATTCGGGCC$\underline{\underline{\text{GCC}}}$CCT$\underline{\underline{\text{GCCGCC}}}$ATCTCCTCC-3′ and 5′- GGAGGAGAT$\underline{\underline{\text{GGCGGC}}}$AGG$\underline{\underline{\text{GGC}}}$GGCCCGAAT-3′ (double underlines indicate the mutation sites) for the PHB2 mutant (R86A, R88A and K89A), 5′-ATAAGAATGCGGCCGCTATGACCACCCCAGGAAAAG-3′ and 5’-CCGCTCGAGCTCATCAAAGCTGGAAACCTTCCATAGGAG-3’ for KPNA1 (single underlines indicate the recognition sites of the restriction enzymes), 5′-ATAAGAATGCGGCCGCTATGTCCACCAACGAGAATGC-3′ and 5’- CCGCTCGAGCCTACTAAAAGTTAAAGGTCCCAGGAGCCC-3’ for KPNA2, 5′-ATAAGAATGCGGCCGCTATGGCCGAGAACCCCAGC-3′ and 5’- CCGCTCGAGCTTATTAAAAATTAAATTCTTTTGTTTGAA-3’ for KPNA3, 5′-ATAAGAATGCGGCCGCTATGGCGGACAACGAGAAAC-3′ and 5’- CCGCTCGAGCCTACTAAAACTGGAACCCTTCTGTTGGTA-3’ for KPNA4, 5′-ATAAGAATGCGGCCGCTATGGATGCCATGGCTAGTCC-3′ and 5’- CCGCTCGAGCTTATTAAAGTTGAAATCCATCCATTGGTG-3’ for KPNA5, and 5′-ATAAGAATGCGGCCGCTATGGAGACCATGGCGAGCC-3′ and 5’- CCGCTCGAGCTTATTATAGCTGGAAGCCCTCCATGGGGG-3’ for KPNA6. The PCR products of PHB2_1-189_ and PHB2_190-299_ were inserted into the *EcoR*I and *Xho*I sites of the pCAGGSnHC expression vector in frame with a hemagglutinin (HA) tag at the C-terminus. The PCR products of each KPNA were inserted into *Not*I and *Xho*I sites of the pCAGGSn3FH expression vector in frame with a FLAG-tag at the N-terminus. The final construct DNA sequences were confirmed via DNA sequencing (ABI3500xL x24; Applied Biosystems, Foster City, CA, USA).

### Interactions between PHB2 and KPNAs

COS-7 cells were plated in 6-well plates at 1 × 10^6^ cells per well and individually co-transfected with and HA-PHB2 and FLAG-KPNAs (KPNA1 to 6) using the FuGENE6 transfection reagent (Promega, Madison, WI, USA) as described previously [8, 9]. For the identification of KPNAs-binding regions in PHB2, three different constructs corresponding to partial PHB2 (PHB2_1–189_, PHB2_190–299_) and NLS mutant PHB2 (R86A, R88A and K89A) were transfected into COS-7 cells, respectively. At 48 h after transfection, the cells were lysed with 0.1% NP-40 lysis buffer. The lysates were immunoprecipitated with anti-FLAG M2 agarose (Sigma) for 12 h at 4°C and were eluted with 3x FLAG-peptide (Sigma) followed by immunoblot analysis as described above.

### Nuclear/cytoplasmic fractionation

MCF-7 cells and COS-7 cells were treated as described above, and the nuclear and cytoplasmic fractions were prepared using the NE-PER nuclear and cytoplasmic extraction reagent (Thermo Fisher Scientific) as described previously [8]. α/β-Tubulin and lamin B1 were used as loading controls for the cytoplasmic and nuclear fractions, respectively.

### Immunocytochemical staining

COS-7 cells were seeded at 1 × 10^5^ cells per well (Laboratory-Tek II Chamber Slide System; Nalge Nunc International, Naperville, IL, USA) under E2-free or 10 nM E2 conditions as described below. The COS-7 cells were co-transfected with HA-PHB2, FLAG-KPNAs (KPNA1, 2, 5 or 6) and FLAG- ERα, and then treated with 10 nM E2 for 24 h. The cells were then fixed with phosphate buffered saline (PBS) containing 4% paraformaldehyde at 4°C for 30 min and rendered permeable with PBS containing 0.1% Triton X-100 at 4°C for 2 min. Subsequently, the cells were covered with 3% BSA in PBS for 1 h to block non-specific hybridization followed by incubation with anti-HA antibody diluted at 1:500 for another 1 h. After washing with PBS, the cells were stained with Alexa 594-conjugated anti-rat secondary antibody (Molecular Probe, Eugene, OR, USA) diluted at 1:1,000 for 1 h. The nuclei were counter-stained with 4′, 6′-diamidine-2′-phenylindole dihydrochloride (DAPI). Fluorescent images were obtained with an Olympus IX71 microscope (Olympus, Tokyo, Japan). The nuclear intensity of translocated PHB2 were calculated using MetaMorph software (Molecular Devices, Tokyo, Japan), and are expressed as the ratio of translocated PHB2 at four different points. For endogenous BIG3 staining, MCF-7 cells were incubated with anti-BIG3 diluted at 1:500 followed by with Alexa 488-conjugated anti-rat secondary antibody.

### Gene silencing via RNA interference

To evaluate the subcellular localization of the PHB2 and *TFF1* expression level in cells in which *BIG3* and *KPNA* gene were knocked down by siRNA, we used siRNA oligonucleotides (Sigma) as described previously [8, 9]. The sequences targeting *KPNA*, *BIG3* or enhanced green fluorescent protein gene (*EGFP*) were as follows: 5’-AAUGUGCUUUCCUGGUUGCUG-3’ for *KPNA1*, 5’-CAGUGUUCCGAGACUUGGUUA-3’ for *KPNA2*, 5’-GAAGCAGCUUGGGCUAUAA-3’ for *KPNA5*, 5’-AACUGUUCCAUCCUUAAUCCU-3’ for *KPNA6*, 5'-GAUGCGUUCUCUGCCACAC-3' for *BIG3*, and 5’-GCAGCACGACUUCUUCAAG-3’ for *EGFP*. MCF7 cells were plated at a density of 6.5 x 10^5^ cells in a 6-well plate or 5 x 10^6^ cells in a 10-cm dish and transfected with the above siRNAs using Lipofectamine RNAiMAX Reagent (Life Technologies) in Opti-MEM medium (Life Technologies) according to the manufacturer’s instructions. After 24 h, the cells were treated with 10 nM E2 for 24 h followed by immunoblotting and real-time RT-PCR. The gene silencing effects of the siRNAs were evaluated via immunoblotting using anti-KPNA and BIG3 antibodies. The *TFF1*, *BIG3*, *KPNA1*, *KPNA2*, *KPNA5* and *KPNA6* expression levels were evaluated via real-time PCR using an ABI PRISM 7500 Real-Time PCR system (Life Technologies) and SYBR Premix Ex Taq (Life Technologies). Each sample was normalized to the *β2-MG* mRNA content, and the results were expressed as the fold increases over the untreated cells (set at 1.0). The data represent the means ± SD of two independent experiments. The primers were as follows: 5′-GCCCTTGAAGCCAATATTCC-3′ and 5′-AGATGGTTTCAGTGGGCTTG-3′ for *BIG3*, 5′-GGCCTCCTTAGGCAAATGTT-3′ and 5′-CCTCCTCTCTGCTCCAAAGG-3′ for *TFF1*, and 5′-AACTTAGAGGTGGGGAGCAG-3′ and 5′-GAGCTGTGCCCATCTTCA-3′ and 5′-CTCGCAAAGCAGGAGAAA-3′ for *KPNA1*, and 5′-TCTGCTTGGGCACTCACT-3′ and 5′-TGCAGGAGCCGAACTAAG-3′ for *KPNA2*, 5′-GGAAATAGAGCTCAGATTCAGGC-3′ and 5′-ACAAAGTGGTTTAATGCAGCCT-3′ for *KPNA5*, and 5′-AGCTGCCTGGGCTCTAAC-3′ and 5′-AGCATCTGCCAGCAAGTC-3′ for *KPNA6*, and 5′-CACAACCATGCCTTACTTTATC-3′ for *β2-MG* as an internal control.

### Inhibition of BIG3–PHB2 interactions by the dominant negative peptide ERAP

The 13 amino acid peptides derived from the PHB2-binding domain of BIG3 (codons 165–177) were covalently linked at the NH_2_ terminus to a membrane-transducing 11 polyarginine sequence (11R) to construct the ERAP peptide [9]. MCF-7 cells were treated with 10 nM E2 ± 10 μM ERAP. BIG3–PHB2 interactions were assessed using co-immunoprecipitation followed by immunoblotting, as described above.

### Identification of interacting regions between PHB2 and BIG3

To identify the BIG3-binding regions of PHB2, HEK293T cells were plated in 6 cm plates at 1 × 10^6^ cells per well and individually co-transfected with two different constructs corresponding to partial PHB2 (PHB2_1–189_, PHB2_190–299_) and BIG3 as described in construction of expression vectors. Forty-eight hours later, the interactions between HA-PHB2 and FLAG-BIG3 were assessed using co-immunoprecipitation followed by immunoblotting, as described above.

### Statistical analysis

Statistical significance was calculated using Student’s *t* test to evaluate gene expression. A difference of *P* < 0.05 was considered statistically significant.

## Results

### Interactions between PHB2 and KPNAs

**Fig 1:**
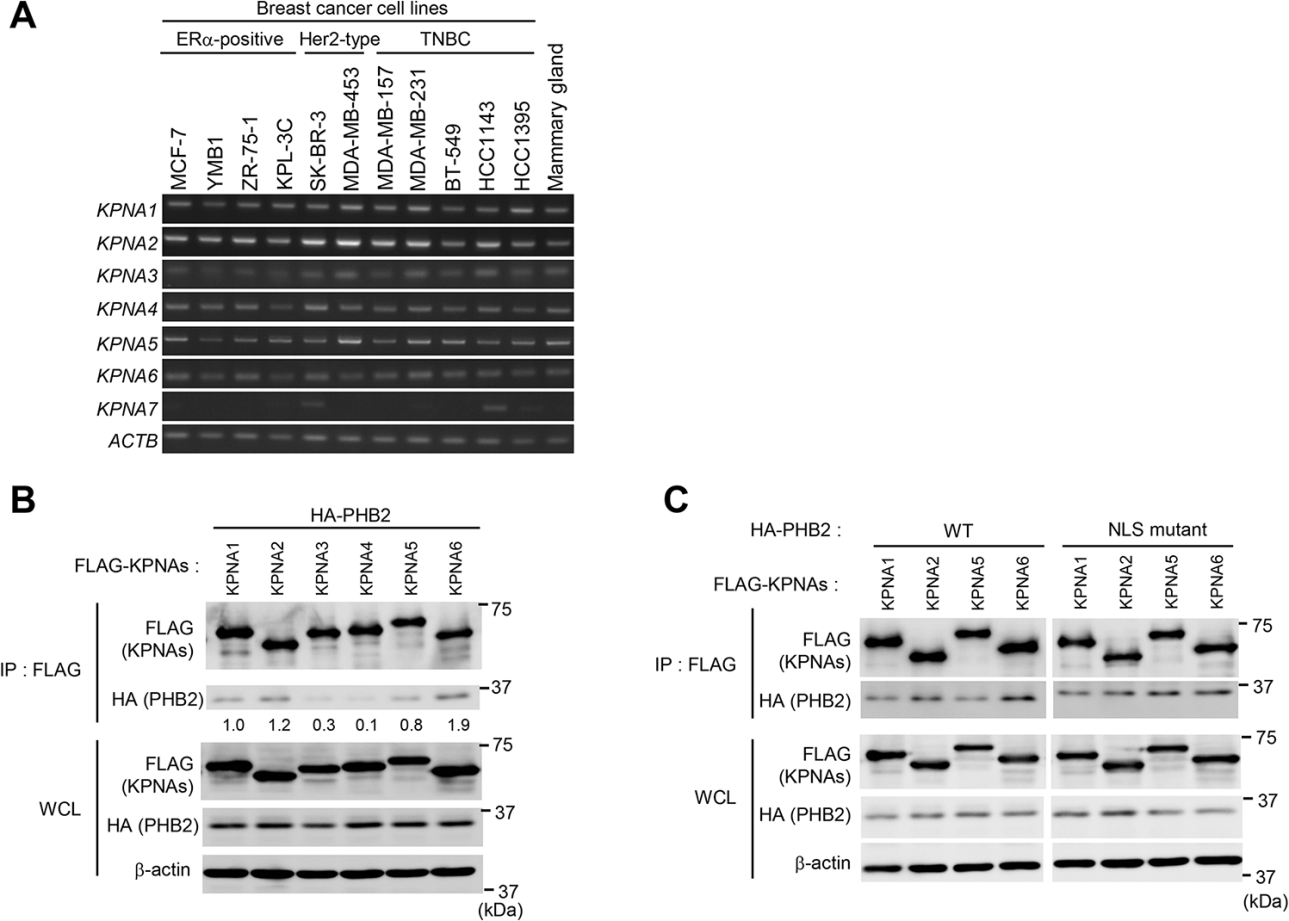
PHB2 interact with KPNAs. (A) The expression levels of the KPNA family of proteins in breast cancer cell lines and normal human mammary gland tissue were evaluated using semi-quantitative RT-PCR. *ACTB* is used as an internal control; (B, C) Immunoblotting analysis was performed to assess the interactions between PHB2 (B) or PHB2 NLS mutants (C) and each KPNA. The lysates from COS-7 cells transfected with PHB2 or PHB2 NLS mutants and each KPNA were immunoprecipitated with anti-FLAG antibody. Full-length images of immunoblots and semi-quantitative RT-PCR are shown in S1A–S1C Fig The data are expressed the fold increase over HA-PHB2 which bound to KPNA1 (set at 1.0) (B).

Because previous reports have shown that PHB2 has a putative nuclear localization signal (NLS) at 86-RPRK-89 [4, 13], we hypothesized that PHB2 is translocated to the nucleus via its interaction with Karyopherin-alpha family (KPNAs, importin-alpha) proteins in breast cancer cells. There are seven known subtypes of KPNA proteins in human cells [11, 17]. We first investigated the endogenous expression of *KPNA*s gene (*KPNA1* to *7*) in 11 breast cancer cell lines and normal mammary gland tissue via semi-quantitative RT-PCR. The results showed that *KPNA1* to *6* was highly expressed in all 11 breast cancer cell lines as well as in normal mammary gland tissue, while focal *KPNA7* expression was observed in the SK-BR-3 and HCC1143 cell lines (Fig 1A). Next, to determine which KPNAs (KPNA1 to 6) interact with PHB2, we constructed plasmids designed to express HA-tagged PHB2 (HA-PHB2) and FLAG-tagged KPNAs (FLAG-KPNA1 to 6) (see Materials and Methods). These plasmids were co-transfected into COS-7 cells, and the proteins were immunoprecipitated with anti-FLAG antibody. Immunoblot analysis of the precipitates using anti-HA antibody indicated that HA-PHB2 was strongly bound to FLAG-KPNA1,-KPNA2,-KPNA5 and-KPNA6 and slightly bound to FLAG-KPNA3 and-KPNA4, respectively (Fig 1B). To determine whether the predicted classic NLS (86-RPRK-89) of PHB2 is required for its nuclear translocation, we generated constructs containing NLS mutants of PHB2 in which three conserved amino acids (R86, R88 and K89) had been substituted with alanine, respectively. Then, HA-tagged PHB2 NLS mutants and FLAG-tagged KPNAs (FLAG-KPNA1,-KPNA2,-KPNA5, and-KPNA6) were co-transfected into COS-7 cells, respectively, and the proteins were immunoprecipitated with anti-FLAG antibody followed by immunoblot analysis with anti-HA antibody. Unexpectedly, these substitutions did not abolish the interactions between PHB2 and any of the KPNA proteins, including FLAG-KPNA1,-KPNA2,-KPNA5, and-KPNA6 (Fig 1C). These findings suggest that PHB2 may interact with KPNAs via a non-NLS or one or more novel NLS sequences.

### KPNA mediates the nuclear translocation of PHB2

**Fig 2:**
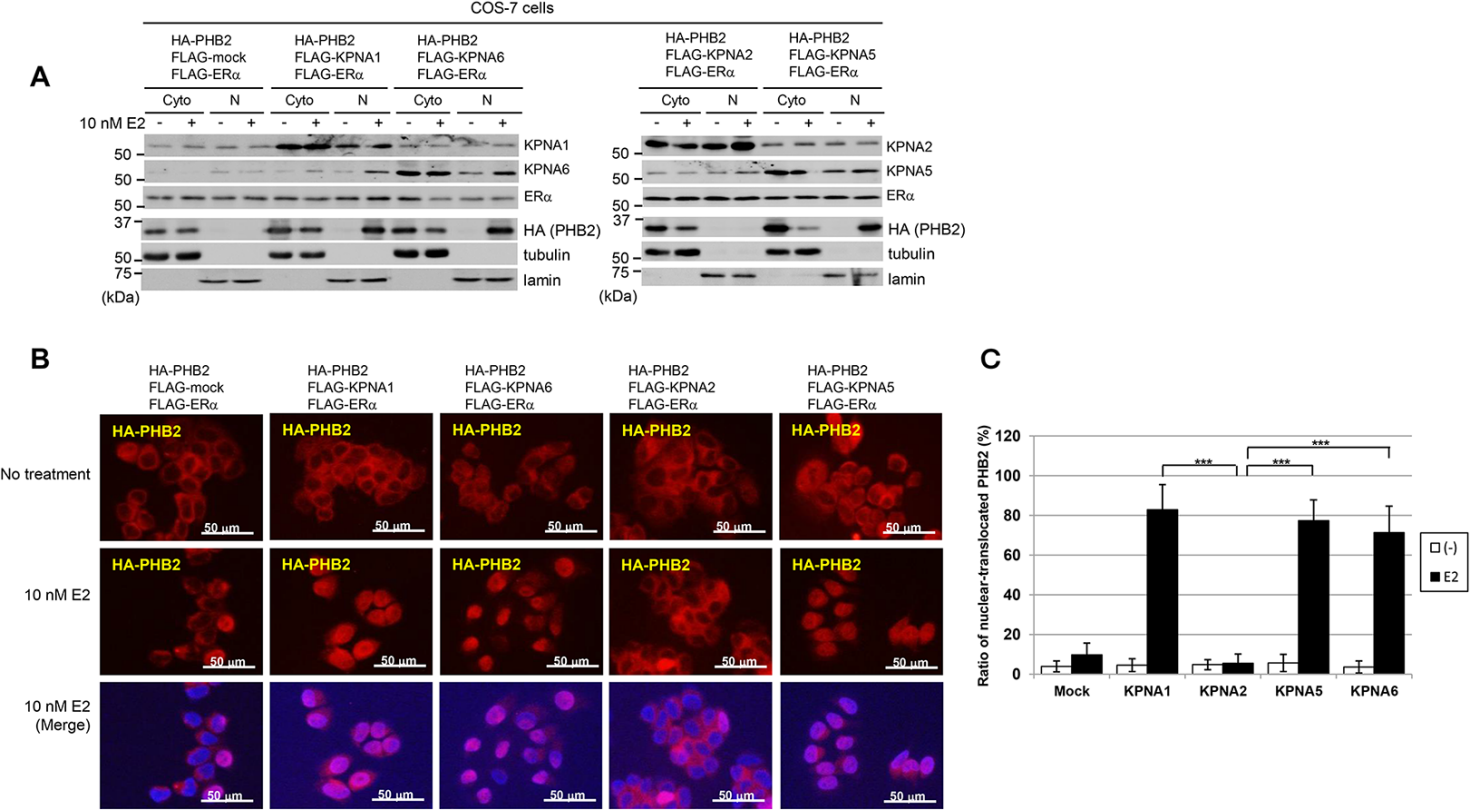
KPNA mediates the nuclear translocation of PHB2. (A) Immunoblotting analysis was performed to detect the subcellular localization of KPNA, ERα and PHB2. COS-7 cells co-transfected with HA-PHB2, each FLAG-KPNA and FLAG-ERα were treated with 10 nM E2 for 24 h and separated into cytoplasmic and nuclear fractions. Each KPNAs and ERα were detected by endogenous antibody. α/β-Tubulin (tubulin) and lamin B1 (lamin) were used as loading controls for the cytoplasmic (Cyto) and nuclear (N) fractions, respectively. (B) Representative immunofluorescence images of the subcellular localization of HA-PHB2 in COS-7 cells are shown; HA-PHB2 (red), DAPI (blue). (C) Statistical analyses of the nuclear intensity of translocated PHB2. The data represent the mean ± SD of four different points (***P<0.001 in a two-sided Student’s *t*-test).

We next investigated the possibility that PHB2 is translocated to the nucleus via the KPNAs in the presence of E2. FLAG-KPNAs (KPNA1, KPNA2, KPNA5, and KPNA6), FLAG-ERα and HA-PHB2 were co-transfected into COS-7 cells, followed (24 h later) by E2 stimulation and immunoblot analysis (see Materials and Methods). The results showed that the overexpression of KPNA1, KPNA5 or KPNA6 but not KPNA2 led to the nuclear translocation of PHB2 in the presence of E2 (Fig 2A). Immunocytochemical staining analysis confirmed that HA-PHB2 localized to the nucleus in the presence of E2 when FLAG-KPNA1,-KPNA5 or-KPNA6 was overexpressed (Fig 2B and 2C). These results suggest that the overexpression of KPNA1, KPNA5, and KPNA6 may induce the nuclear translocation of PHB2 in the presence of E2 and ERα in mammals. However, overexpression of KPNA2 did not. Although we cannot rule out the possibility of KPNA2 as a candidate carrier protein for PHB2 nuclear translocation, in this study we focused on KPNA1, KPNA5, and KPNA6 in further analyses to elucidate the mechanism of PHB2 nuclear translocation via these KPNAs.

### Interactions between endogenous PHB2 and KPNAs in breast cancer cells

**Fig 3:**
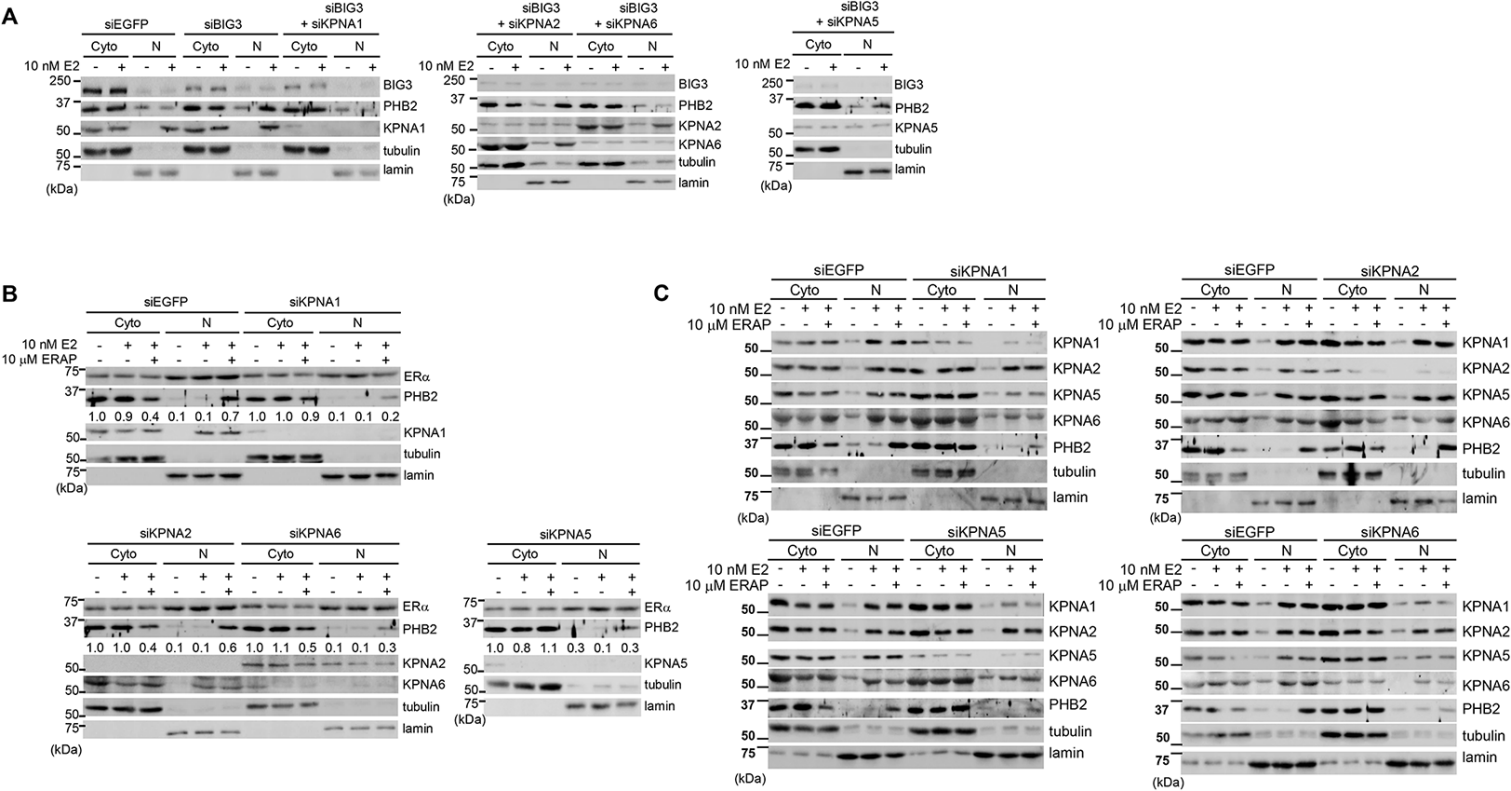
KPNA1, KPNA5, and KPNA6 are required for E2-dependent PHB2 nuclear import in breast cancer cells. (A) Immunoblotting analysis was performed to evaluate the subcellular localization of endogenous PHB2 in the BIG3- and KPNAs (KPNA1, KPNA5, and KPNA6)-depleted MCF-7 cells. MCF-7 cells were treated with siBIG3 and each siKPNA, followed by E2 ± ERAP for 24 h. Then, the cells were separated into cytoplasmic and nuclear fractions; (B) The nuclear translocation of PHB2 in KPNA-depleted MCF-7 cells in the presence of E2 and ERAP was evaluated. MCF-7 cells were treated with each siKPNA followed by E2 ± ERAP for 24 h. Then, the cells were separated into cytoplasmic (Cyto) and nuclear (N) fractions. The data are expressed the fold increase over cytoplasm fraction of untreated siEGFP, siKPNA1, siKPNA2, siKPNA5 or siKPNA6-transfected cells (set at 1.0), respectively. (C) The relationship among each KPNA was evaluated in KPNA-depleted MCF-7 cells in presence of E2 and ERAP. α/β-Tubulin (tubulin) and lamin B1 (lamin) were used as loading controls for the cytoplasmic (Cyto) and nuclear (N) fractions, respectively (A, B). Full-length images of immunoblots are shown in S3A–S3C Fig.

To further validate the KPNA-mediated E2-dependent nuclear translocation of endogenous PHB2 in breast cancer cells, we examined the knockdown effect of each *KPNA* via siRNA treatment on the subcellular distribution of endogenous PHB2 in MCF-7 cells after *BIG3* knockdown followed by E2 treatment. In the presence of E2, the knockdown of *KPNA1*, *KPNA5* and *KPNA6* led to a remarkable decrease in the amount of PHB2 in the nuclei of the BIG3-depleted cells, respectively; however, the knockdown of *KPNA2* did not (Fig 3A). Subsequently, we evaluated the possibility of the ERα-dependent nuclear translocation of PHB2 in the presence of E2 in breast cancer cells as previously reported [18]. We first examined the nuclear translocation of ERα and PHB2 after treatment with E2 and ERAP, a dominant-negative peptide inhibitor that inhibits BIG3-PHB2 interactions [8] in MCF-7 cells. We observed that ERAP led to a decrease in cytoplasmic PHB2, thereby substantially increasing the amount of nuclear PHB2 in the presence of E2 in EGFP siRNA-transfected cells (Fig 3B), but did not change the amount of ERα protein in either the cytoplasm or nuclei of EGFP siRNA-transfected cells regardless of treatment with either E2 or ERAP. Moreover, the knockdown of *KPNA1*, *KPNA2*, *KPNA5*, and *KPNA6* had no effect on the nuclear translocation of ERα in comparison with that of PHB2 (Fig 3B). We examined the expression of each KPNA in cytoplasm and nuclear fractions of each KPNA-depleted MCF-7 cells. The results showed that knocking down of KPNA1, KPNA5 and KPNA6, but not KPNA2, led to abolish PHB2 nuclear-translocation (Fig 3C). Furthermore, knocking down of KPNA1 and KPNA6 also led to abolish nuclear-translocation of all of KPNAs, and depletion of KPNA5 led to moderately reduction of nuclear-translocation of all of KPNAs. These findings suggest that the nuclear import of PHB2 released from BIG3 by siRNA or ERAP is mediated by KPNA1, KPNA5 and KPNA6 in an E2-dependent manner in breast cancer cells.

**Fig 4:**
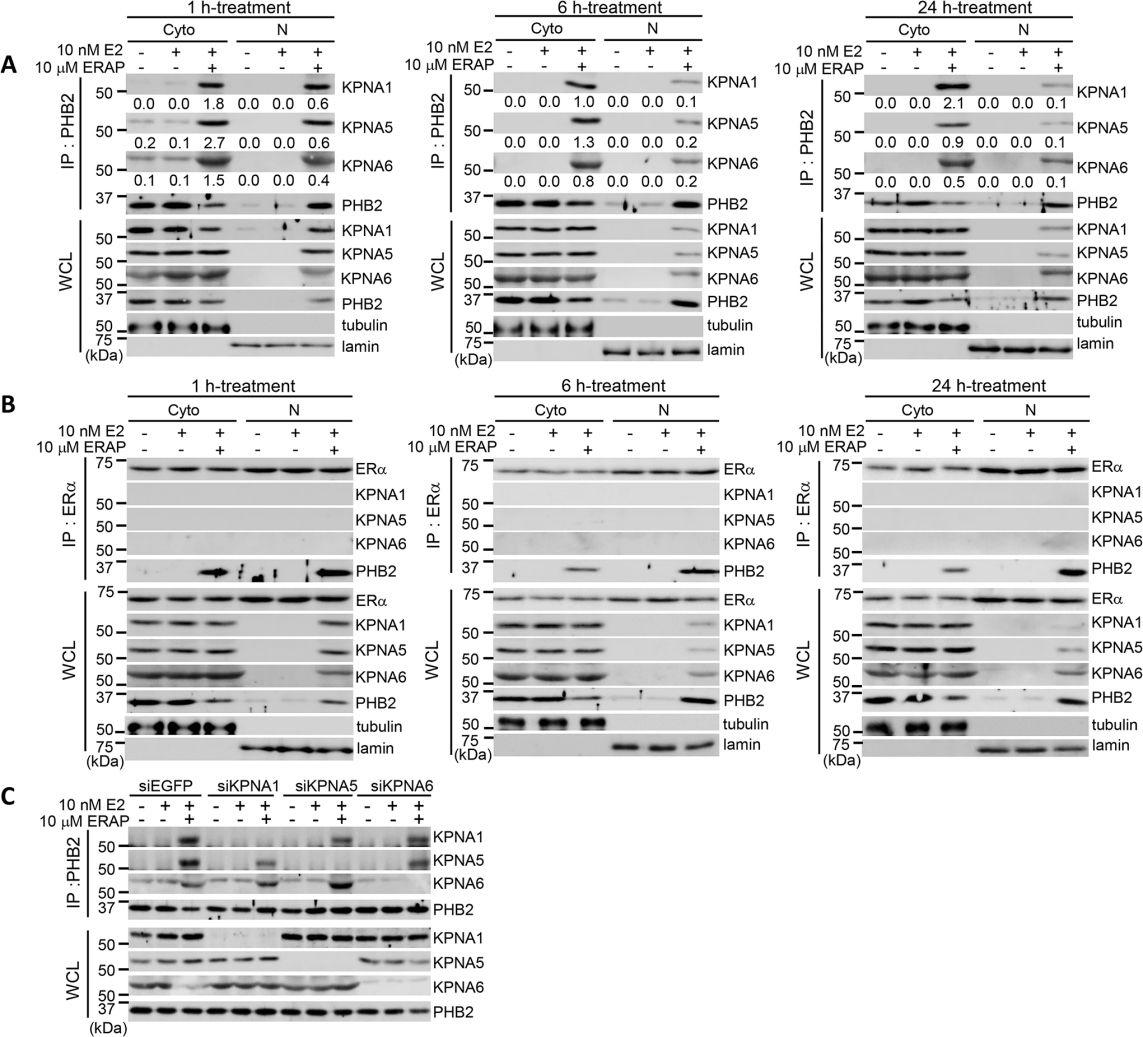
PHB2 interacts with KPNAs, followed by the rapid E2-dependent nuclear translocation in breast cancers. (A, B) Immunoblotting analysis was performed to evaluate the interaction between PHB2 and each KPNA. MCF-7 cells were treated with E2 ± ERAP for 1h (left), 6h (middle) and 24 h (right), respectively, followed by fraction into the cytoplasm and nucleus. Then, each fraction was immunoprecipitated with anti-PHB2 (A) and anti-ERα antibodies (B), respectively, and then immunoblotted with antibodies against the indicated proteins. α/β-Tubulin (tubulin) and lamin B1 (lamin) were used as loading controls for the cytoplasmic (Cyto) and nuclear (N) fractions, respectively. The numbers indicates the intensity ratio of co-immunoprecipitated KPNA with PHB2 to the immunoprecipitated PHB2 in each fraction; (C) The interactions between PHB2 and the KPNAs were evaluated in the presence of E2 and ERAP. MCF-7 cells were treated with siKPNAs followed by E2 ± ERAP for 24 h. The lysates were then immunoprecipitated with anti-PHB2 antibody and immunoblotted with antibodies against each KPNA. Full-length images of immunoblots are shown in S4A–S4C Fig.

We next investigated whether the endogenous PHB2 forms a complex with KPNA1, KPNA5, or KPNA6 in the nuclear or cytoplasmic fractions of MCF-7 cells after ERAP treatment by co-immunoprecipitation experiments with PHB2 and ERα antibodies. In the presence of E2, ERAP treatment led to a remarkable increase the interactions of PHB2 with KPNA1, KPNA5, and KPNA6 in the cytoplasm and nucleus even after 1h (Fig 4A). In addition, in the presence of E2, the amount of nuclear KPNAs (KPNA1, KPNA5, and KPNA6) which bound to PHB2 was gradually decreased after ERAP treatment in a time-dependent fashion (Fig 4A), suggesting that PHB2 released from BIG3 by ERAP treatment rapidly interacts with KPNAs (KPNA1, KPNA5, and KPNA6) in the cytoplasm, thereby leading to its rapid nuclear translocation, followed by its binding to nuclear ERα. On the other hand, KPNAs (KPNA1, KPNA5, and KPNA6) did not co-immunoprecipitate with nuclear ERα even after ERAP treatment (Fig 4B), suggesting that KPNAs could be detached from PHB2 immediately after nuclear import of PHB2. More importantly, we also demonstrated that each PHB2-KPNA complex had no effect when only one of the three KPNAs (KPNA1, KPNA5, or KPNA6) was knocked down (Fig 4C), suggesting that the E2-dependent nuclear translocation of endogenous PHB2 is required for its binding to multiple KPNAs (KPNA1, KPNA5, and KPNA6) in breast cancer cells. These findings indicated that PHB2 released from BIG3 by ERAP rapidly interacts with multiple KPNAs (KPNA1, KPNA5, and KPNA6) in the cytoplasm and that this is followed by KPNA-mediated nuclear translocation in the presence of the E2 stimulus.

### KPNAs-mediated PHB2 inhibits nuclear ERα transactivation in breast cancer cells

**Fig 5:**
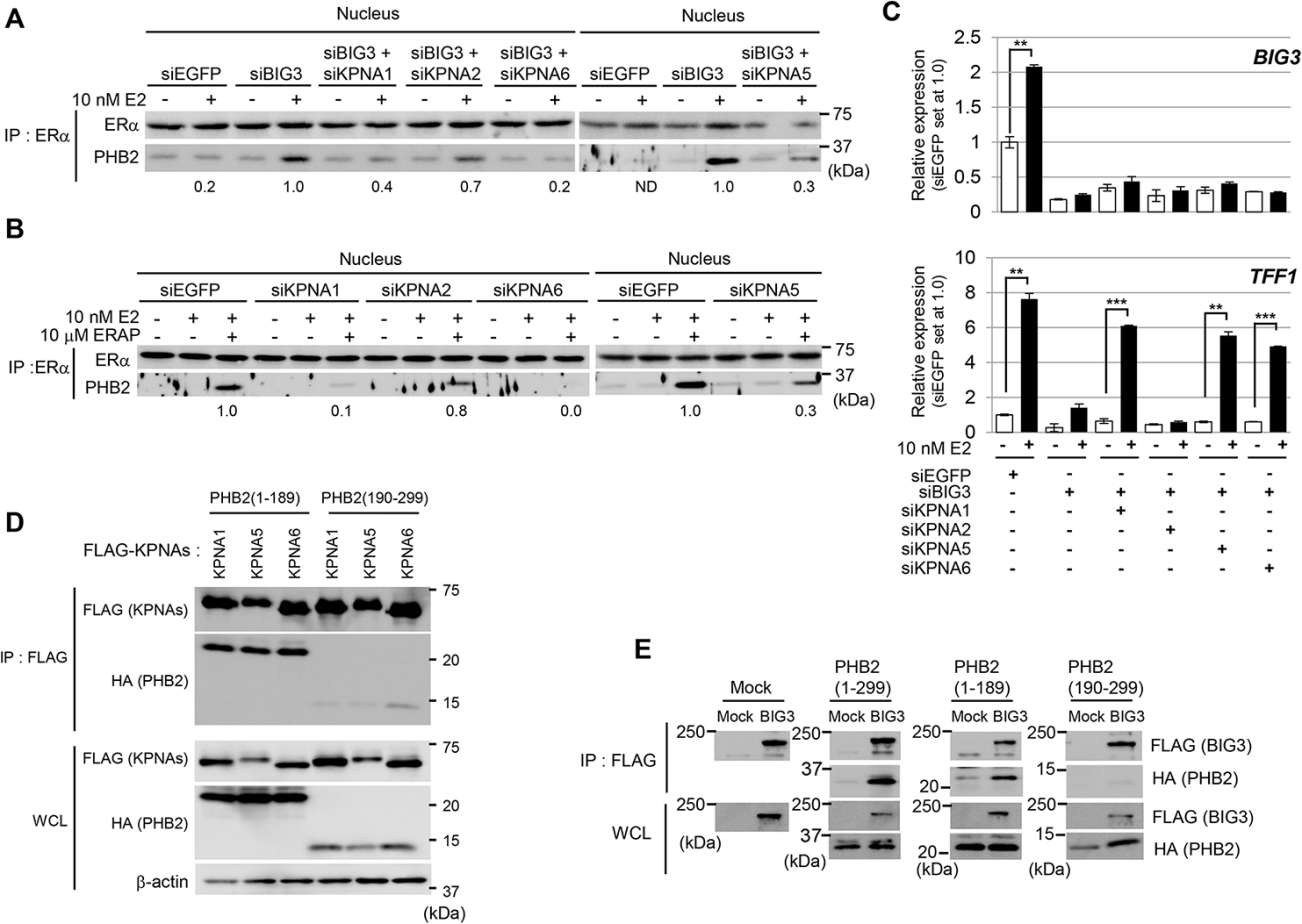
KPNA1, KPNA5, and KPNA6 induce E2-dependent nuclear translocation of PHB2. (A) Immunoblotting analysis was performed to evaluate the interactions between ERα and PHB2 in BIG3- and KPNA (KPNA1, KPNA5, and KPNA6)-depleted MCF-7 cells. MCF-7 cells were treated with siBIG3 and each siKPNA, followed by E2 ± ERAP for 24 h. Then, the nuclear fractions were immunoprecipitated with anti-ERα antibody and were immunoblotted with antibodies against the indicated proteins. The data are expressed the fold increase over E2-treated siBIG3-transfected cells of right and left panels, respectively (set at 1.0). ND: not detected. This experiment was performed using the nuclear fractions used in Fig 3A; (B) The interaction between ERα and PHB2 released by E2 and ERAP in the nuclear fractions was evaluated. MCF-7 cells depleted of each KPNA were treated with E2 ± ERAP for 24 h, and the nuclear fractions were immunoprecipitated with anti-ERα antibody. The data are expressed the fold increase over E2-treated siEGFP-transfected cells of right and left panels, respectively (set at 1.0). This experiment was performed using the nuclear fractions used in Fig 3B; (C) The *TFF1* expression levels following treatment with siBIG3 and siKPNA were evaluated using real-time PCR. The data are expressed as the fold increase over the untreated cells (set at 1.0) and represent the means ± SD of two independent experiments (**P<0.01, ***P<0.001 in a two-sided Student’s *t*-test); (D) Immunoblotting analysis was performed to identify the KPNA-binding regions in PHB2. The lysates from COS-7 cells transfected with the indicated HA-PHB2 constructs and FLAG-KPNAs were immunoprecipitated with an anti-FLAG antibody; (E) Immunoblotting analysis was performed to identify the BIG3-binding region in PHB2. The lysates from HEK293T cells transfected with the indicated HA-PHB2 constructs and FLAG-BIG3 were immunoprecipitated with an anti-FLAG antibody. Full-length images of immunoblots are shown in S6A–S6D Fig.

Our previous reports showed that intrinsic PHB2 released from BIG3 by ERAP directly binds to both nuclear- and membrane-associated ERα [8]. We validated the knockdown effect of each KPNA (KPNA1, KPNA5, and KPNA6) on the interactions between endogenous PHB2 and nuclear ERα in BIG3-depleted cancer cells. The results showed that the depletion of only BIG3 led to interactions between endogenous PHB2 released from BIG3 with nuclear ERα but that the depletion of BIG3 and KPNA1, KPNA5, and KPNA6 did not (Fig 5A), indicating that PHB2 binding to nuclear ERα in cancer cells is KPNA-mediated. Similar results were observed with ERAP treatment in the presence of E2 in KPNA-depleted cells, respectively (Fig 5B). Next, to elucidate the effect of each KPNA on nuclear ERα transcriptional activity, we knocked down *BIG3* and all KPNAs and examined the expression of *TFF1* (an ERα-target gene) via qRT-PCR. We confirmed the upregulation of both the *BIG3* and *TFF1* genes (which have been identified as ERα target genes) in E2-stimulated MCF-7 cells (Fig 5C; siEGFP). By contrast, the knockdown of *BIG3* expression led to the significant suppression of E2-induced *TFF1* expression, whereas knocking down KPNA1, KPNA5, and KPNA6 caused a remarkable up-regulation of *TFF1* expression in BIG3-depleted MCF-7 cells, respectively (Fig 5C, S5 Fig). Taken together, our data clearly demonstrate that KPNA1, KPNA5, and KPNA6 primarily regulate the E2-dependent nuclear translocation of endogenous PHB2 released from BIG3 in the presence of E2.

### Binding regions of PHB2 and each KPNA

To determine the binding regions of PHB2 and each KPNA, we constructed plasmids designed to express HA-PHB2 deletion mutants—HA-PHB2 (1–189) and (190–299), respectively—and FLAG-KPNAs (FLAG-KPNA1, KPNA5, and KPNA6) (see Materials and Methods). These plasmids were co-transfected into COS-7 cells, and the proteins were then immunoprecipitated with anti-FLAG antibody. Immunoblot analysis of the precipitates using anti-HA antibody indicated that HA-PHB2 (1–189) but not HA-PHB2 (190–299) was bound strongly to all FLAG-KPNA1,-KPNA5, and-KPNA6 proteins (Fig 5D). Notably, we demonstrated that HA-PHB2 (1–189) but not HA-PHB2 (190–299) was also bound to the FLAG-BIG3 protein (Fig 5E). Taken together, these data raised the possibility that BIG3 structurally overlay the KPNAs (KPNA1, KPNA5, and KPNA6) binding region(s) of PHB2 (excluding the ERAP binding region), leading to the inhibition of KPNA-mediated PHB2 nuclear translocation in the presence of E2 in breast cancer cells and resulting in constitutive E2-dependent ERα transcriptional activity.

## Discussion

**Fig 6:**
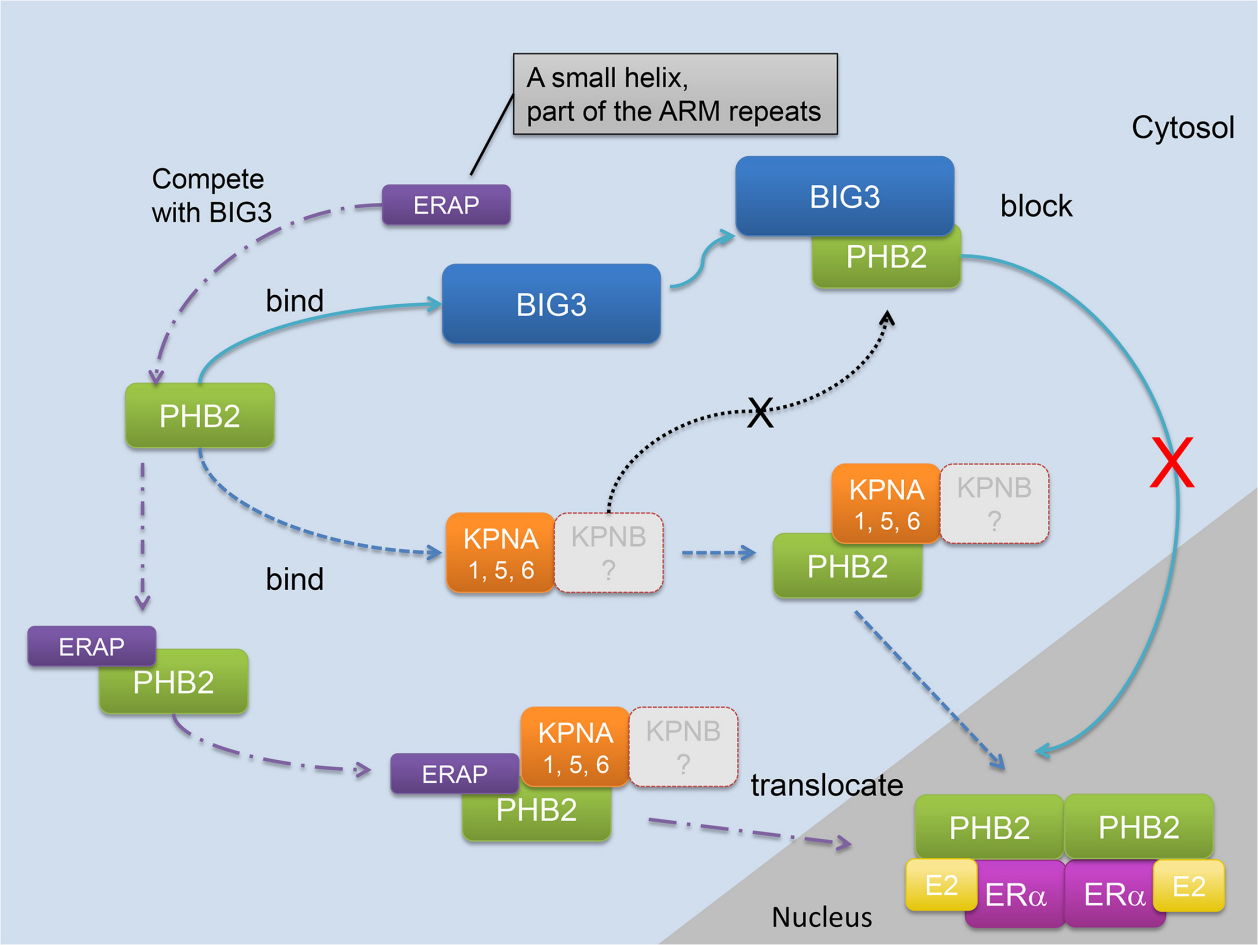
The mechanism of the E2-dependent nuclear translocation of PHB2 through KPNAs in breast cancer cells.

Previous studies have shown that PHB2 directly binds to nuclear ERα, resulting in suppression of ERα transcriptional activity by competing with the co-activator SRC-1 to bind ERα [5] and by recruiting histone deacetylase 1 [HDAC1; ref. 6] and a co-repressor, NcoR [5, 6], in breast cancer cells, suggesting that PHB2 acts as a co-repressor of ERα. However, this is controversial because endogenous PHB2 is abundantly expressed in ERα-positive breast cancer cells. Therefore, it was unclear how PHB2 is inactivated in ERα-positive cancer cells. Our previous studies demonstrated that endogenous BIG3 mainly localized in cytoplasm (S7 Fig) and sequesters PHB2, thereby causing the loss of function of PHB2 and resulting in constitutive ERα transcriptional activation in breast cancer cells. This information suggests that the BIG3-PHB2 complex plays a critical role in promoting ERα-positive breast cancer cell growth [8–10]. However, the mechanism by which BIG3 blocks E2-dependent PHB2 nuclear translocation in breast cancer cells remains unclear. In this study, we demonstrated that KPNAs (KPNA1, KPNA5, and KPNA6) primarily interact with PHB2 released from BIG3 via siRNA or ERAP treatment in the cytoplasm and that this is followed by KPNA-mediated nuclear translocation in the presence of E2 stimulation. Notably, endogenous PHB2 preferentially bound to BIG3 in MCF-7 cells regardless of the abundant presence of KPNAs, suggesting the possibility that endogenous PHB2 has a high affinity to BIG3 protein compared with that to KPNAs in breast cancer cells. In addition, when KPNA1, KPNA5, or KPNA6 was knocked down by siRNA, the inhibition of PHB2 nuclear translocation occurred in the presence of E2 in BIG3-depleted cells, indicating that all three (KPNA1, KPNA5, and KPNA6) are required for the nuclear translocation of PHB2. Interestingly, human KPNA1, KPNA5, and KPNA6 are classified into the same group by their amino acid sequences and share a minimum 80.7% identity [17, 19, 20]. On the other hand, overexpression of only KPNA1, KPNA5, or KPNA6 also led to PHB2 nuclear translocation in COS-7 cells (Fig 2A and 2B). A possible reason for this discrepancy is due to that each KPNA may interact with PHB2 through a mutual, complementary, or reciprocal relationship in overexpression experiments. Moreover, although nuclear proteins are generally known to be transported into the nucleus by forming complexes with KPNA and KPNB heterodimers [11, 12], we found only nuclear-translocation of PHB2 via KPNAs. Therefore, further analyses are needed to elucidate the involvement of endogenous KPNB in PHB2 nuclear-translocation in breast cancer cells (Fig 6). Furthermore, we demonstrated that overexpression of KPNA2 interacts with PHB2 but is not responsible for PHB2 nuclear translocation (Fig 1B and 1C, Fig 2A and 2B). Accumulating evidence indicates that KPNA2 upregulation is significantly associated with poor prognosis in various human cancers [21–24]. Notably, cytoplasmic KPNA2 may have an oncogenic role by binding to the cytoplasmic tumor suppressor NBS1, which is involved in the PI3K/Akt signaling pathway. Accordingly, these evidences indicate the possibility that the role of KPNA2 binding to PHB2 may be independent of nuclear transport in breast cancer cells. Although further analyses are also needed to elucidate the detailed mechanisms of this process, when taken together, these findings and ours suggest that KPNA1, KPNA5, and KPNA6 are likely involved in the sufficient nuclear import of functional PHB2 proteins.

Another interesting finding of this study is that PHB2 possibly interacts with KPNAs via its non-NLS sequence(s) despite bearing a putative nuclear localization signal (NLS; 86-RPRK-89), as described previously [4, 13]. The binding capability of PHB2 with each KPNA was comparable with that of the Ala-replaced NLS mutant PHB2 (R86A, R88A and K89A) (Fig 1C). Notably, the scores of NLS sequences within PHB2 predicted using the SeqNLS algorithm [25] and cNLS Mapper [26] were substantially less than the cut-off value (data not shown). In recent years, however, it has become increasingly apparent that a number of proteins do not follow these canonical pathways and instead utilize non-conventional mechanisms [27]. We also noted the potential of non-conventional nuclear import pathways due to the complicated binding structure of PHB2 and the KPNAs.

However, we determined the KPNAs binding region(s) within the 1 to 189th amino acid region of PHB2 through biochemical analyses (Fig 5D). Furthermore, we identified several candidate protein binding sites within the 1 to 189th amino acid region of PHB2 using the PSIVER (Protein-protein interaction SItes prediction serVER) software [28]. More importantly, our biochemical analysis data demonstrated that PHB2 interacts with BIG3 via its N-terminal portion as well as KPNAs (KPNA1, KPNA5, and KPNA6) (Fig 5E). Furthermore, we previously demonstrated that a dominant-negative peptide, ERAP, based on the residues Q165, D168 and Q173 in BIG3, which are essential for its hererodimerization with PHB2, competitively inhibits BIG3-PHB2 interactions [8]. According to these observations, we can propose that the nuclear translocation of PHB2 is mediated by KPNA (KPNA1, KPNA5, and KPNA6) binding via non-NLS or novel NLS sequence(s) within its N-terminal portion (with the exception of ERAP-binding amino acids) (Fig 5E). Taken together, these findings suggest that BIG3 structurally overlays each KPNA binding region of PHB2, thereby blocking PHB2-KPNA interactions and resulting in the inhibition of E2-dependent PHB2 nuclear translocation in breast cancer cells (Fig 6). Further studies to clarify the BIG3 and KPNA (KPNA1, KPNA5, and KPNA6)-binding regions of PHB2 are now underway.

In conclusion, our findings are the first to show that multiple KPNAs (KPNA1, KPNA5, and KPNA6) play key roles in PHB2 nuclear translocation in the presence of E2 stimulation. In breast cancers, however, BIG3 captures PHB2 through its KPNA (KPNA1, KPNA5, and KPNA6)-binding region(s), thereby inhibiting the E2-dependent suppressive ability of PHB2. These findings may shed light on currently unrecognized ERα signaling networks in breast carcinogenesis. Clarifications of the relationship among BIG3, PHB2, and the KPNAs (KPNA1, KPNA5, and KPNA6) will inform the development of novel therapeutic agents targeting protein-protein interactions, thereby enhancing the nuclear import of the tumor suppressor.

## Supporting Information

S1 Fig A, Full-length of images of semi-quantitative RT-PCR of Fig 1A. B, Full-length of images of all immunoblots of Fig 1B. C, Fig Full-length of images of all immunoblots of Fig 1C.
(PDF)

S2 Fig Full-length of images of all immunoblots of Fig 2A. (TIF)

S3 Fig A, Full-length of images of all immunoblots of Fig 3A. B, Full-length of images of all immunoblots of Fig 3B. C, Fig Full-length of images of all immunoblots of Fig 3C.
(PDF)

S4 Fig A, Full-length of images of all immunoblots of Fig 4A. B, Full-length of images of all immunoblots of Fig 4B. C, Fig Full-length of images of all immunoblots of Fig 4C.
(PDF)

S5 Fig The knockdown effects of si*KPNAs* on *KPNAs* expression in MCF-7 cells. The expression of *KPNAs* were evaluated by real-time PCR. The data are expressed as the fold increase over the untreated cells (set at 1.0) and represent the means ± SD of two independent experiments (*P<0.05, **P<0.01, ***P<0.001) in a two-sided Student’s *t*-test.
(TIF)

S6 Fig A, Full-length of images of all immunoblots of Fig 5A. B, Fig Full-length of images of all immunoblots of Fig 5B. C, Full-length of images of all immunoblots of Fig 5D. D, Full-length of images of all immunoblots of Fig 5E.
(PDF)

S7 Fig Representative immunofluorescence images of the subcellular localization of BIG3. BIG3 (green), DAPI (blue).
(TIF)
