# Supplementary figures and images for: BIG3 Inhibits the Estrogen-Dependent Nuclear Translocation of PHB2 via Multiple Karyopherin-Alpha Proteins in Breast Cancer Cells
Source: PLoS One. 2015 Jun 8;10(6):e0127707. doi: 10.1371/journal.pone.0127707 (PMC4460025; doi:10.1371/journal.pone.0127707)

A

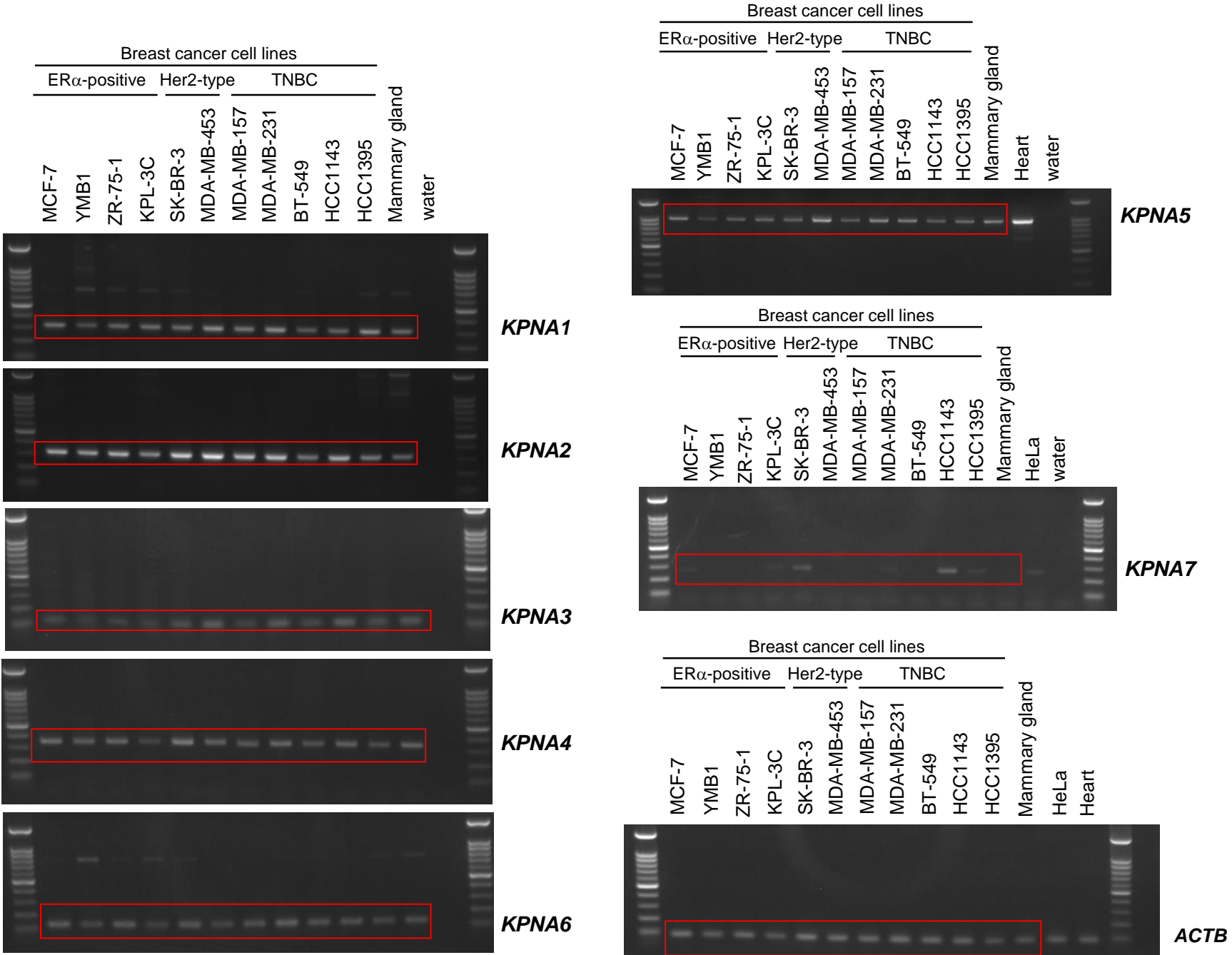

B

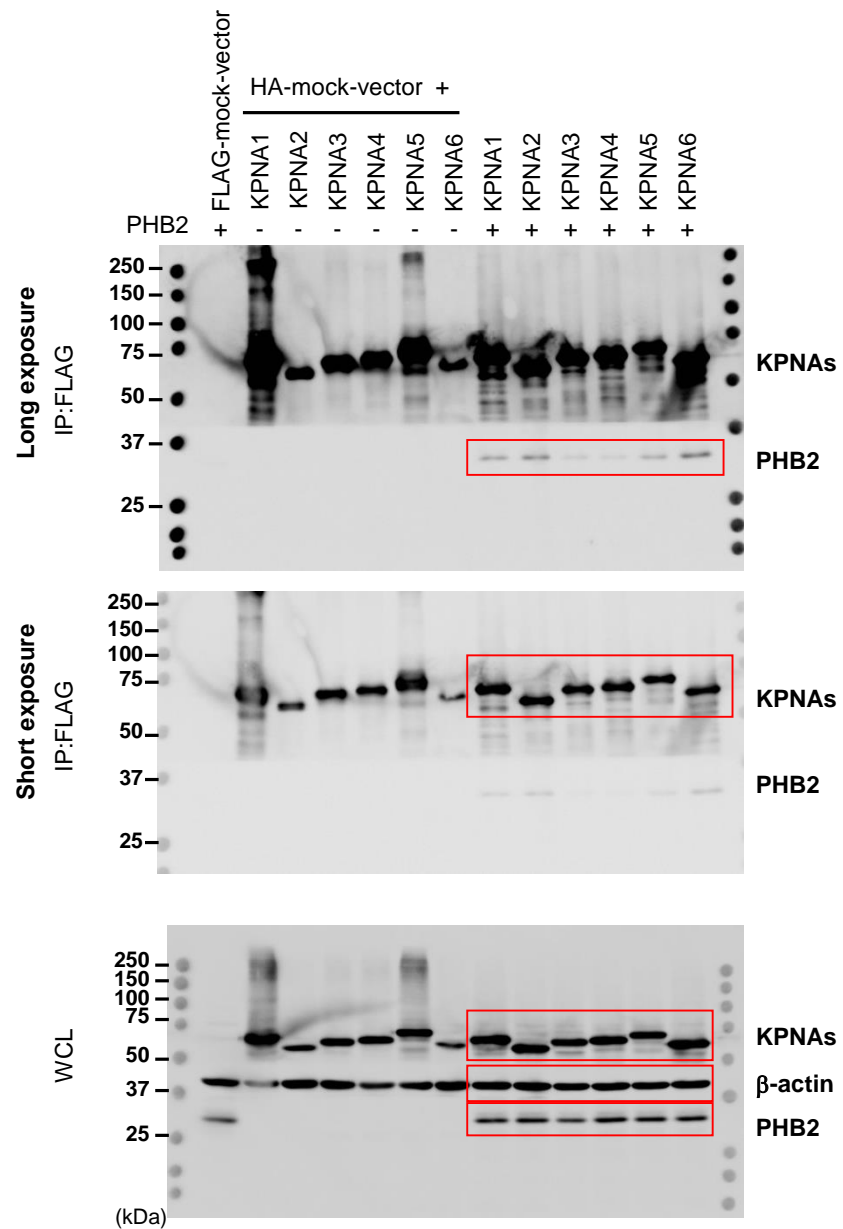

C

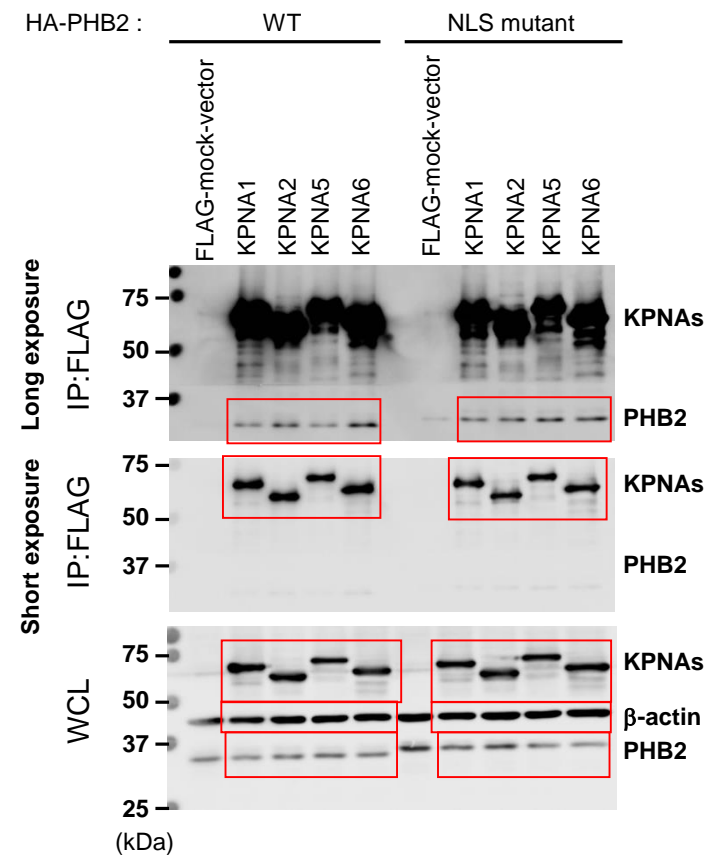

Supplement: S1 Fig — B, Full-length of images of all immunoblots of Fig 1B. C, Fig Full-length of images of all immunoblots of Fig 1C. (PDF) [file pone.0127707.s001.pdf]

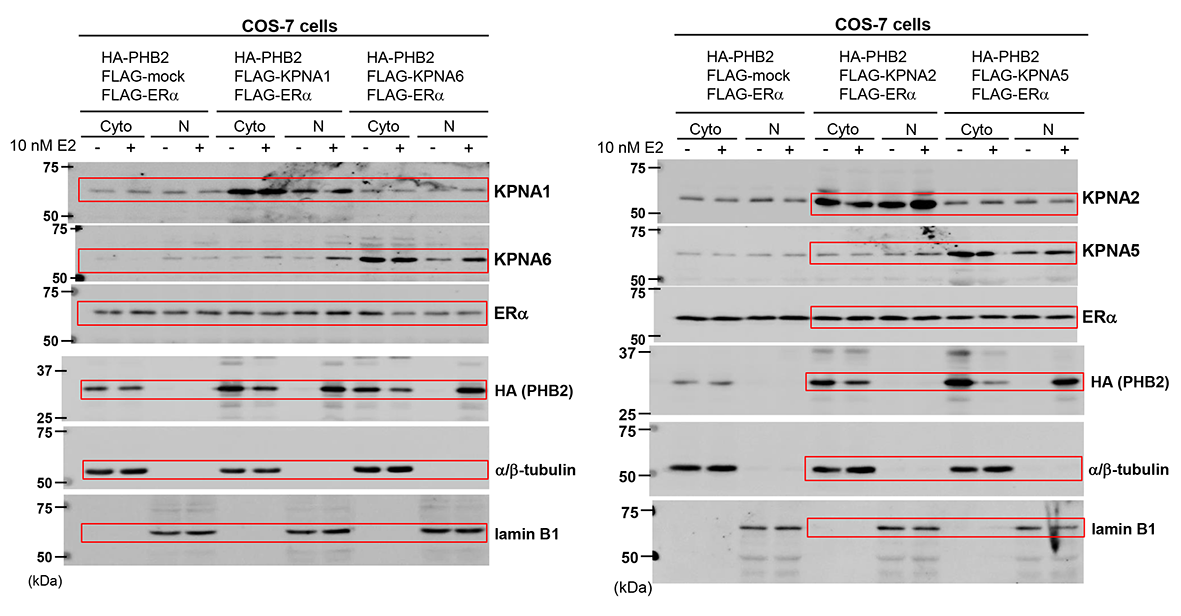

Supplement: S2 Fig — (TIF) [file pone.0127707.s002.tif]

A

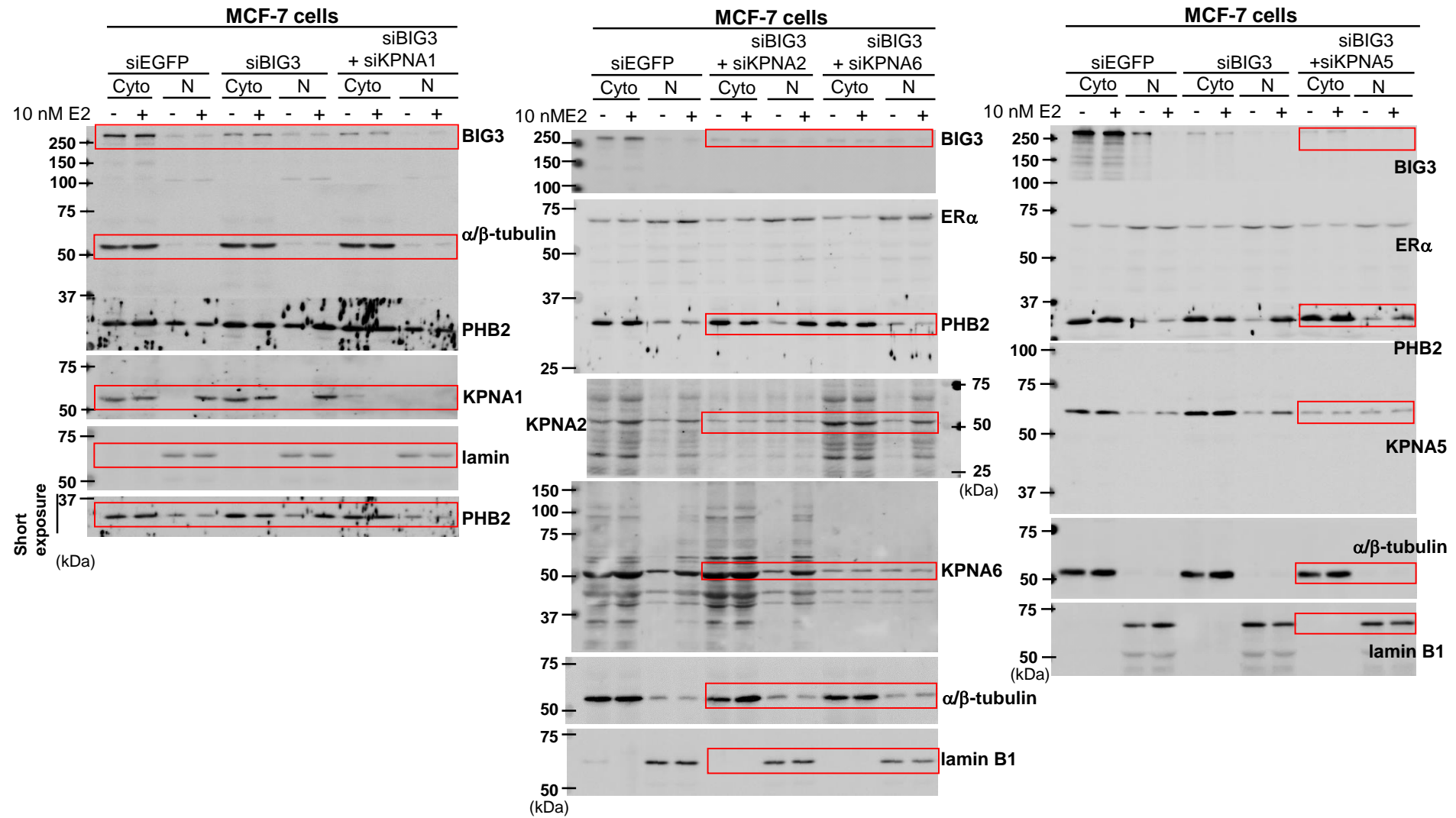

MCF-7 cells

B

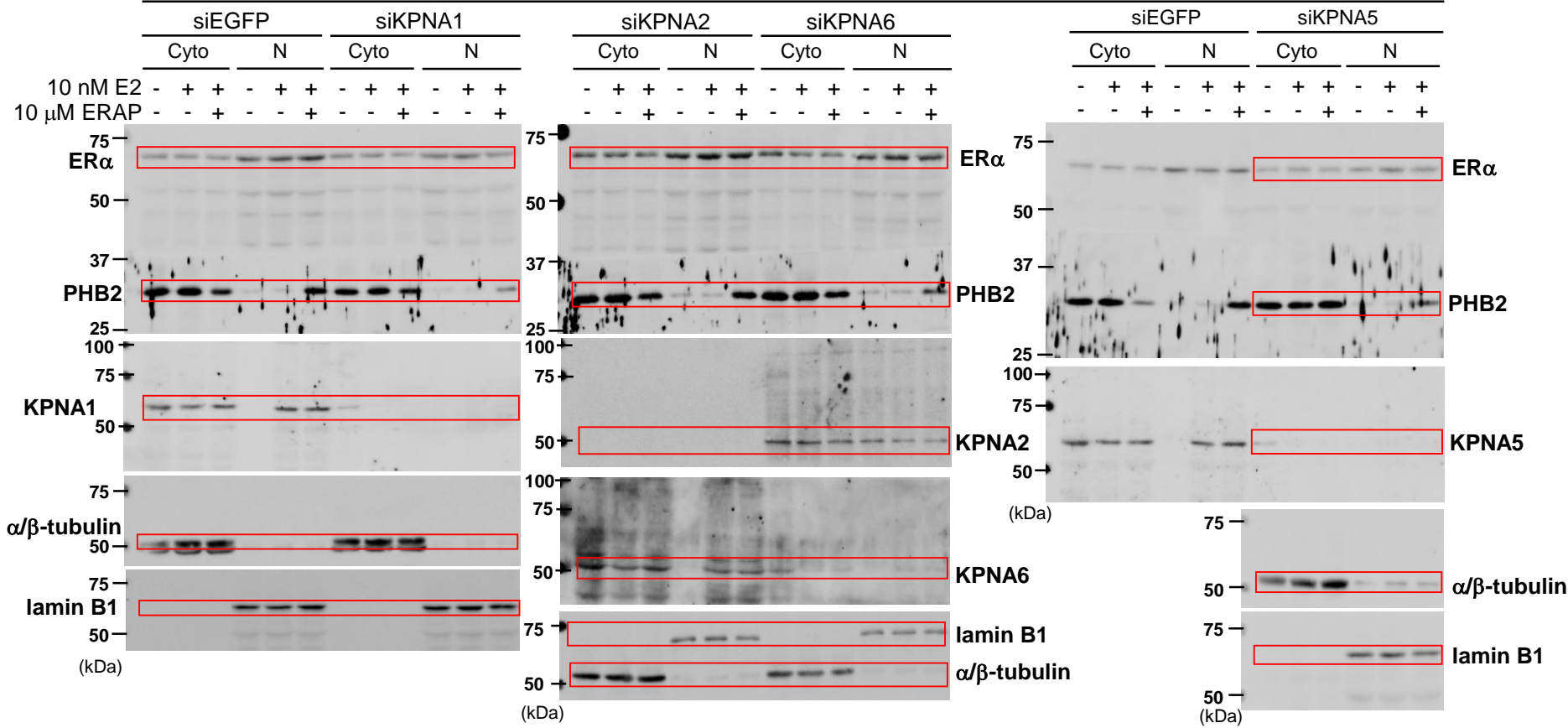

C

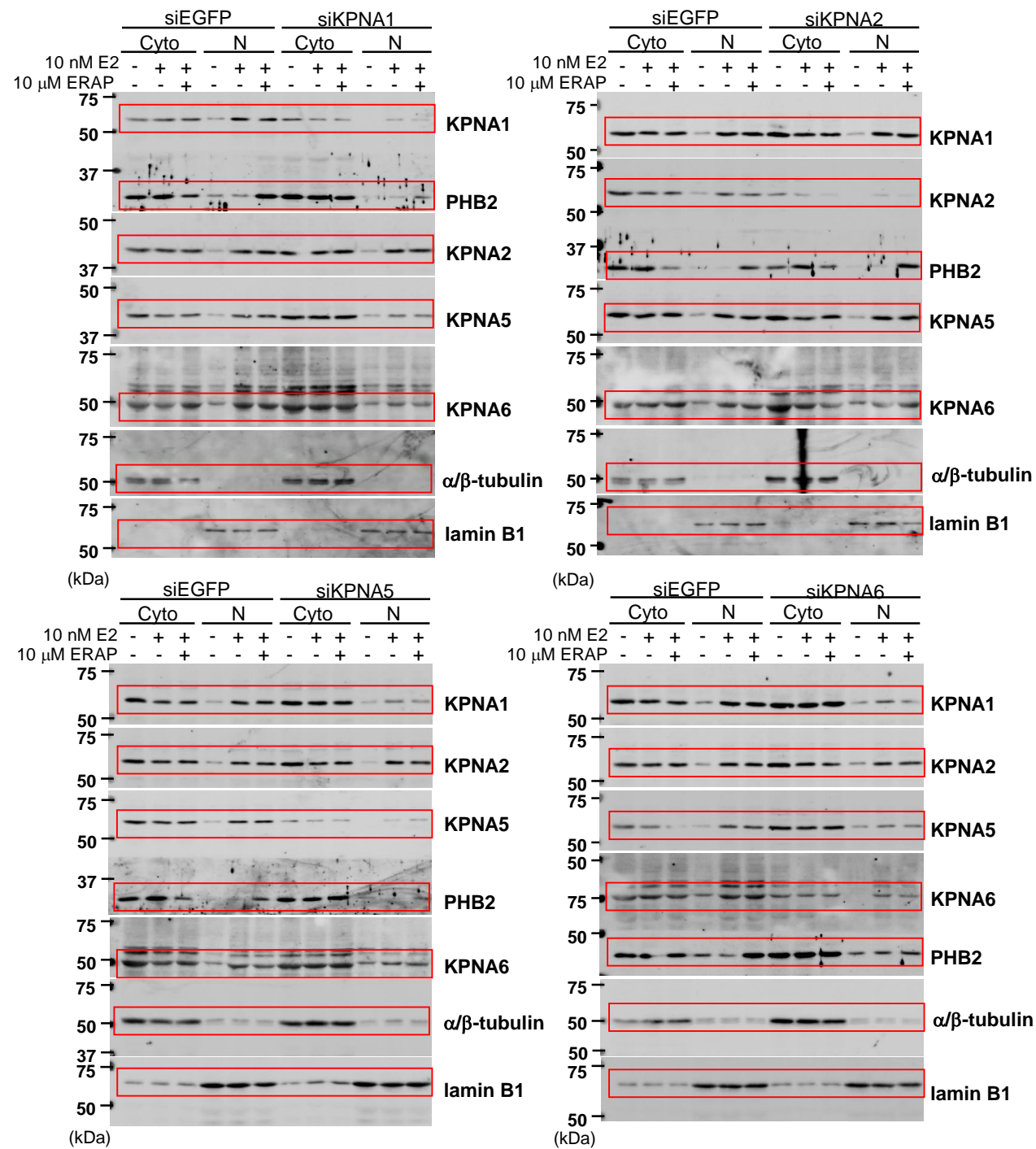

Supplement: S3 Fig — B, Full-length of images of all immunoblots of Fig 3B. C, Fig Full-length of images of all immunoblots of Fig 3C. (PDF) [file pone.0127707.s003.pdf]

## A

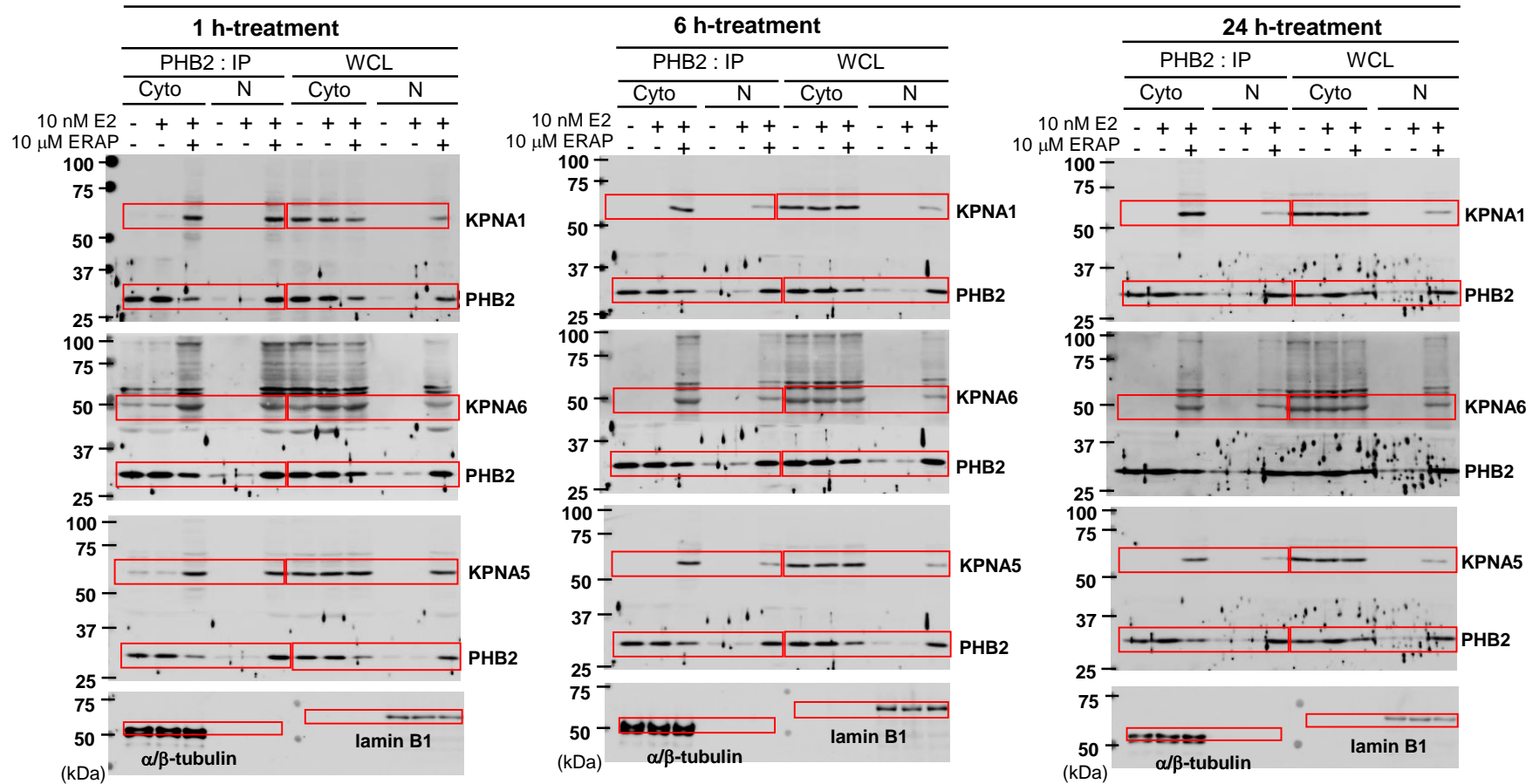

B

MCF-7 cells

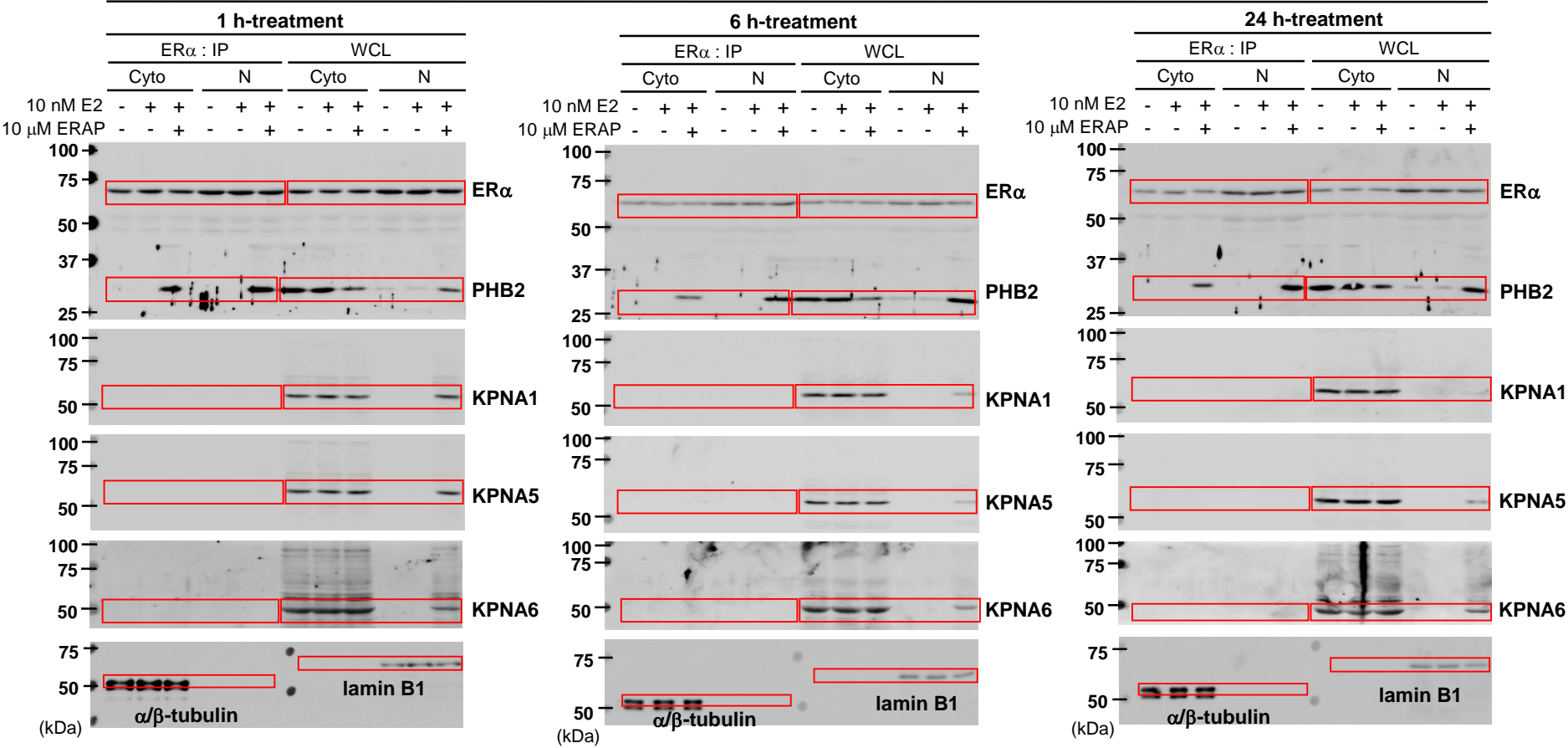

C

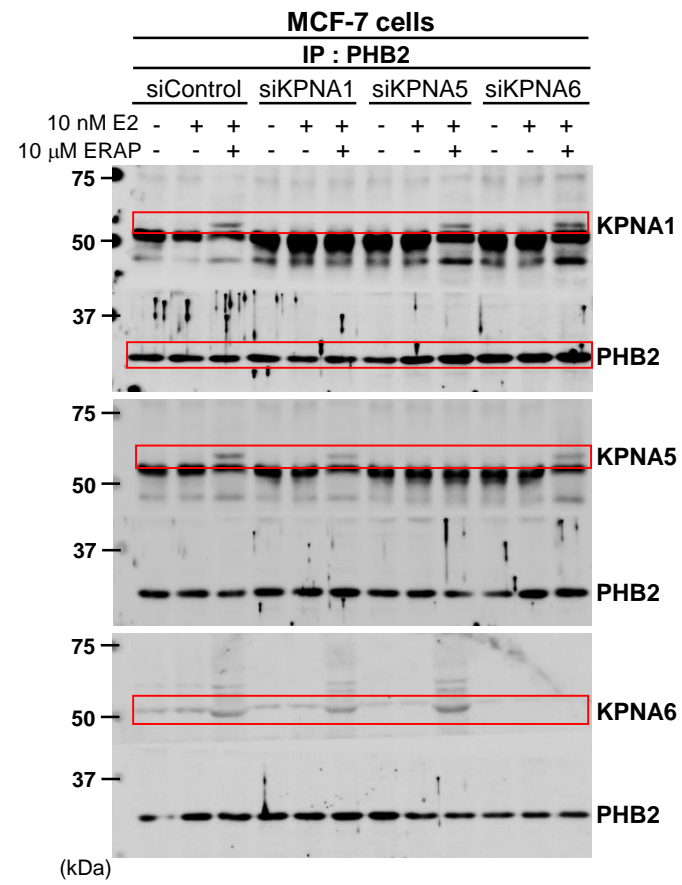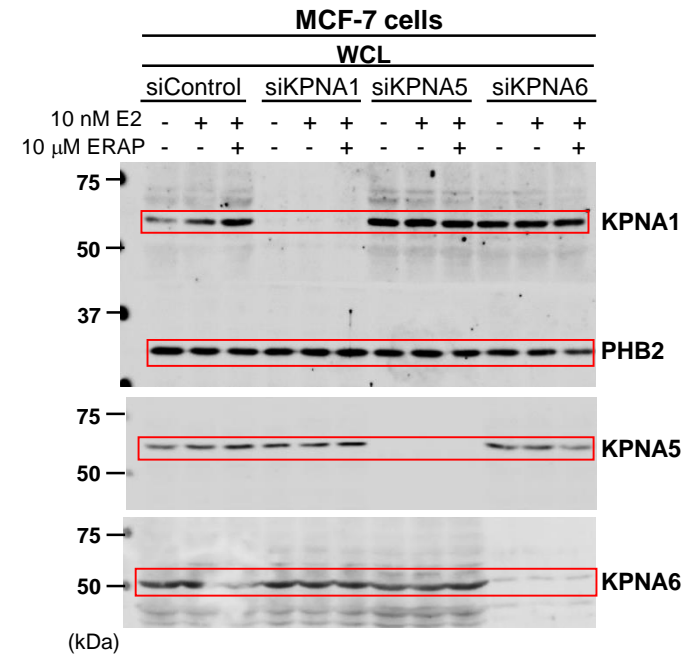

Supplement: S4 Fig — B, Full-length of images of all immunoblots of Fig 4B. C, Fig Full-length of images of all immunoblots of Fig 4C. (PDF) [file pone.0127707.s004.pdf]

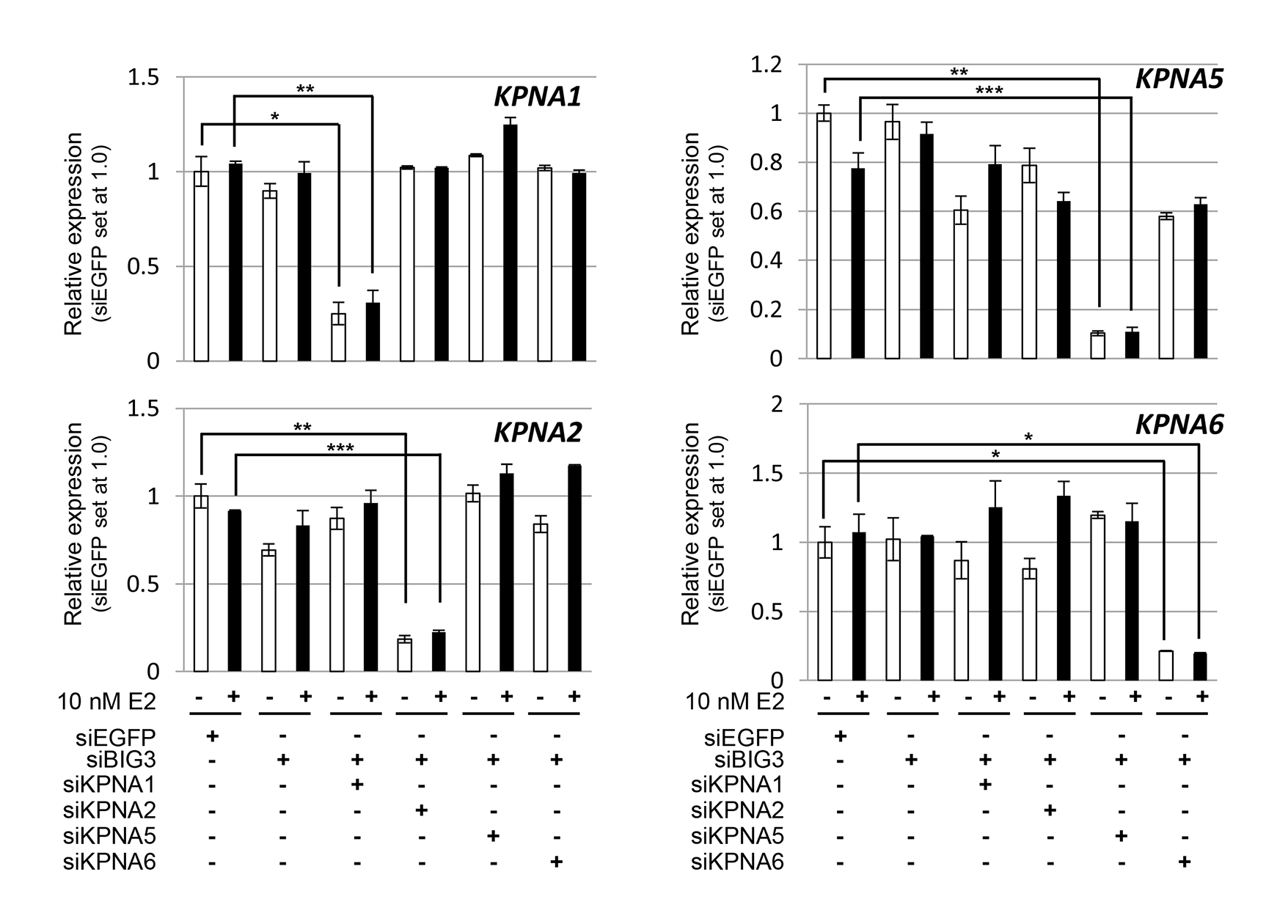

Supplement: S5 Fig — The expression of KPNAs were evaluated by real-time PCR. The data are expressed as the fold increase over the untreated cells (set at 1.0) and represent the means ± SD of two independent experiments (*P<0.05, **P<0.01, ***P<0.001) in a two-sided Student’s t-test. (TIF) [file pone.0127707.s005.tif]

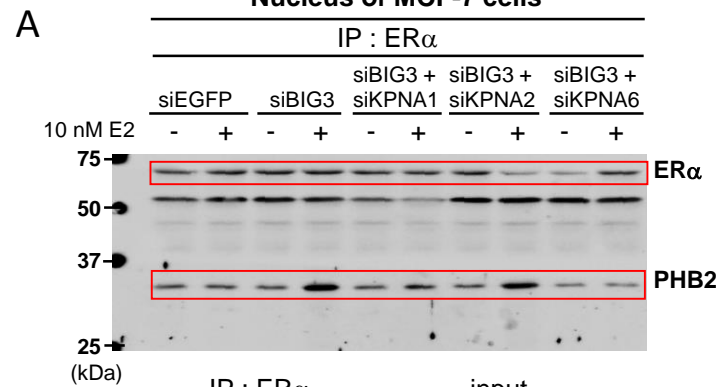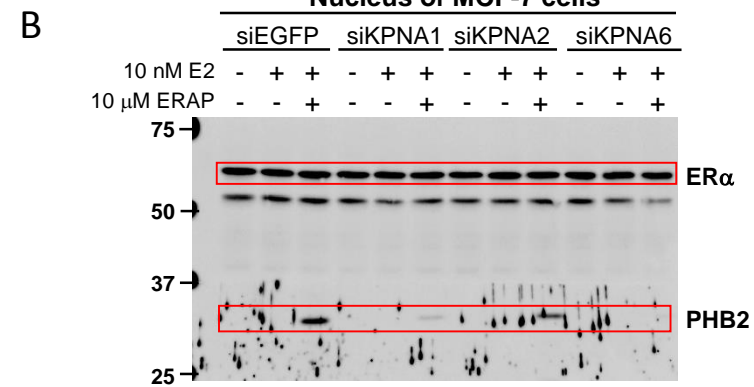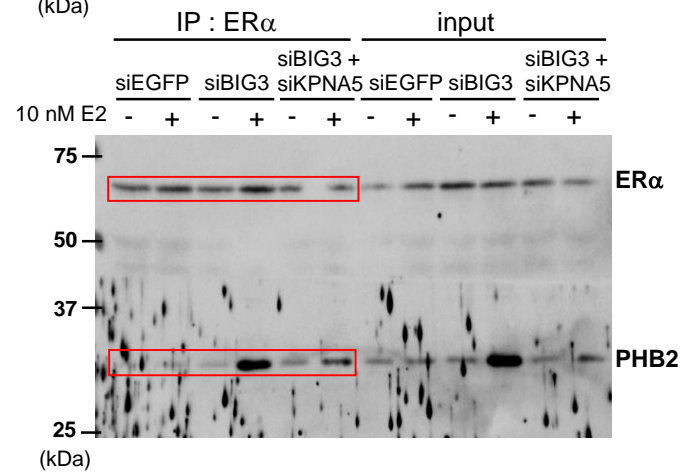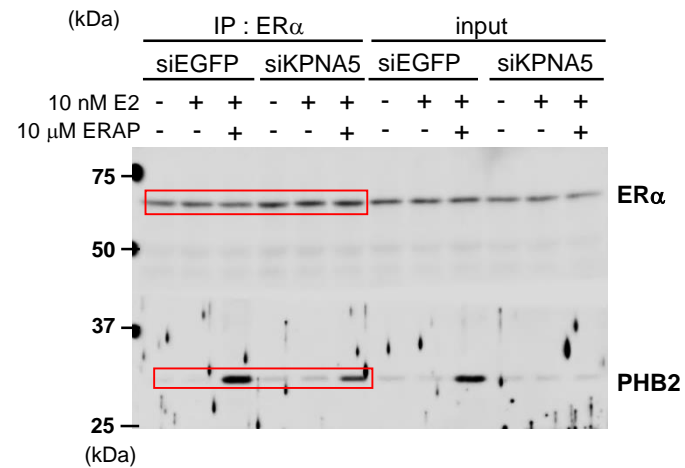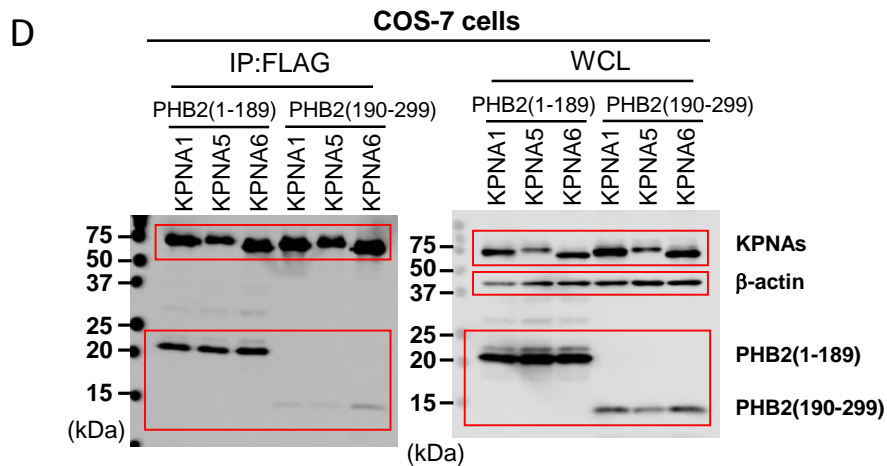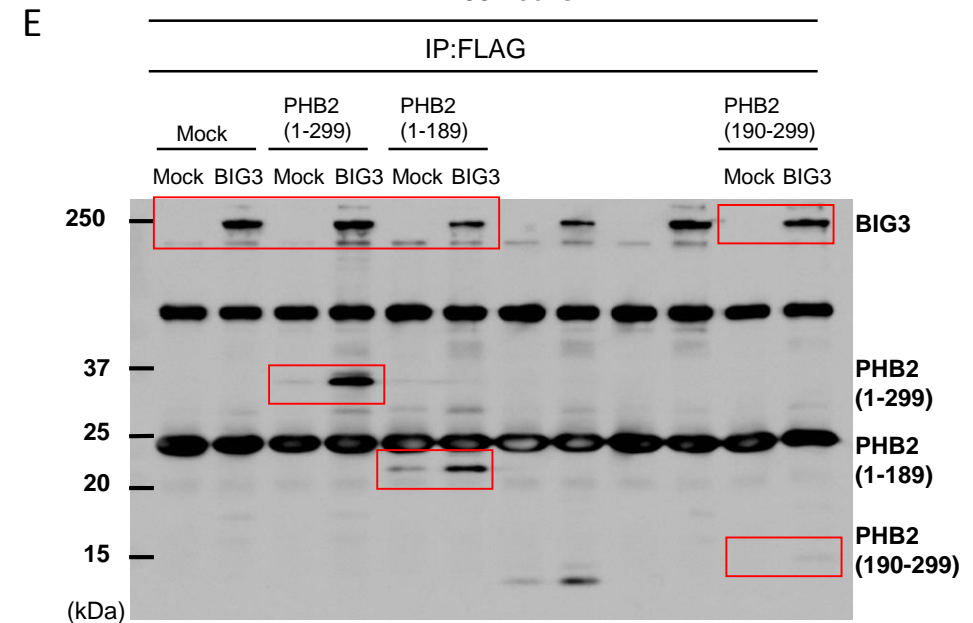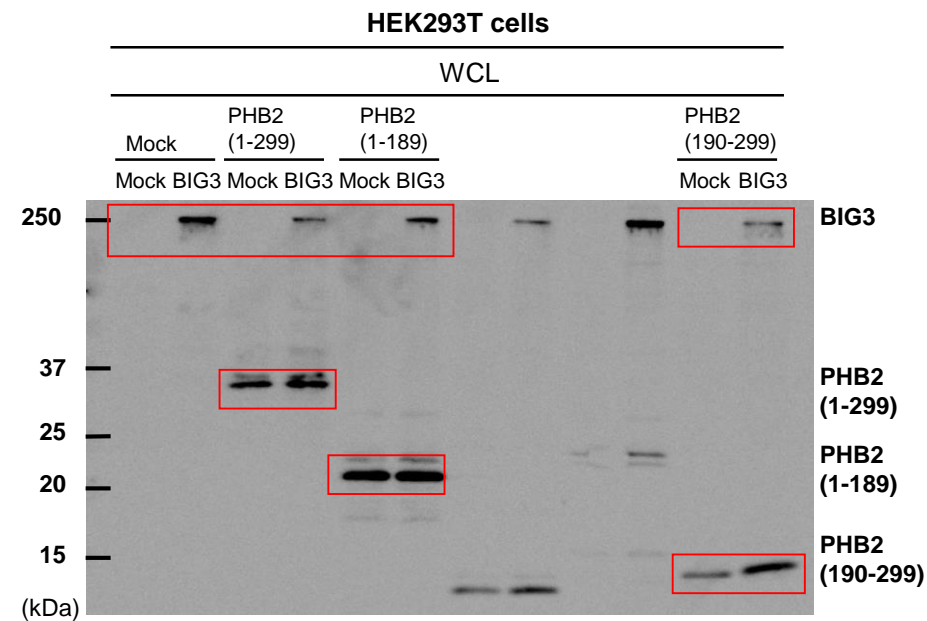

Supplement: S6 Fig — B, Fig Full-length of images of all immunoblots of Fig 5B. C, Full-length of images of all immunoblots of Fig 5D. D, Full-length of images of all immunoblots of Fig 5E. (PDF) [file pone.0127707.s006.pdf]

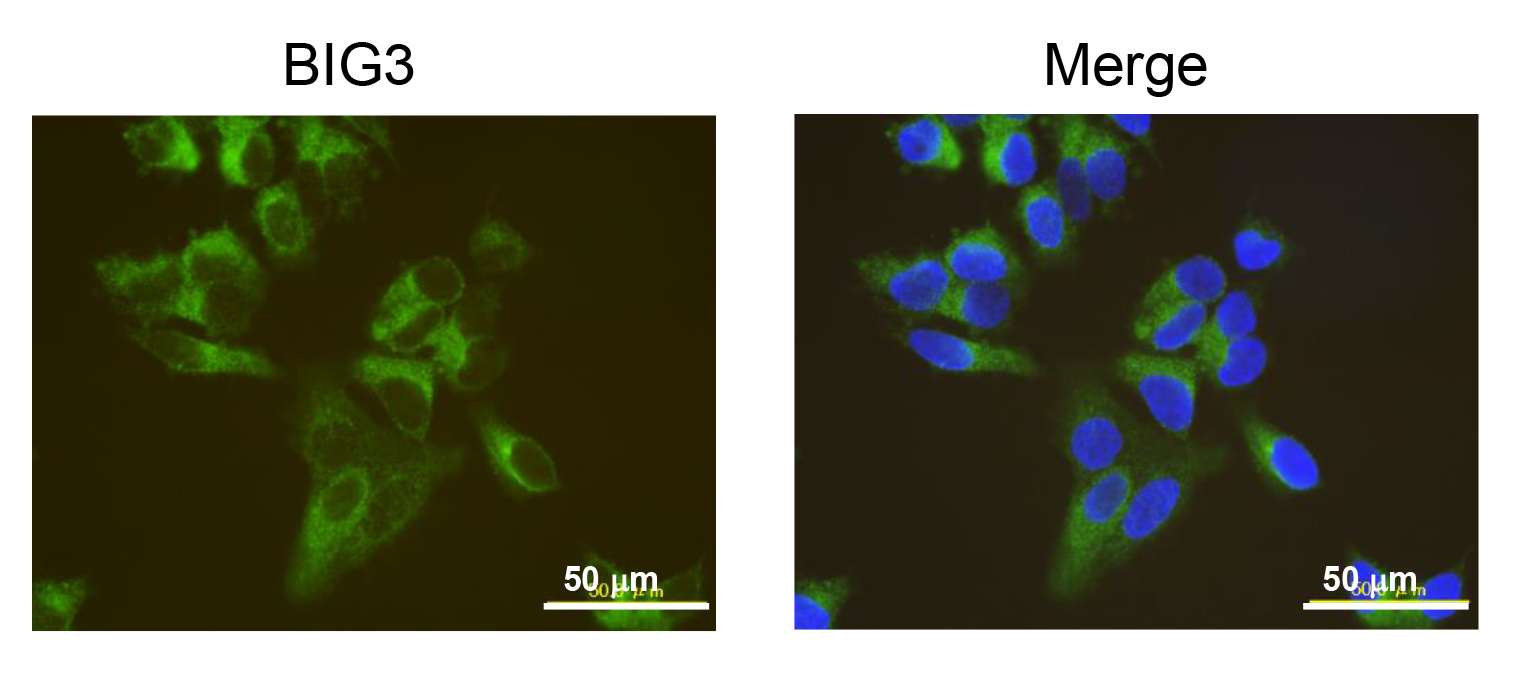

Supplement: S7 Fig — BIG3 (green), DAPI (blue). (TIF) [file pone.0127707.s007.tif]
